# Geographically Structured Populations of *Cryptococcus neoformans* Variety *grubii* in Asia Correlate with HIV Status and Show a Clonal Population Structure

**DOI:** 10.1371/journal.pone.0072222

**Published:** 2013-09-03

**Authors:** Kantarawee Khayhan, Ferry Hagen, Weihua Pan, Sitali Simwami, Matthew C. Fisher, Retno Wahyuningsih, Arunaloke Chakrabarti, Anuradha Chowdhary, Reiko Ikeda, Saad J. Taj-Aldeen, Ziauddin Khan, Margaret Ip, Darma Imran, Ridhawati Sjam, Pojana Sriburee, Wanqing Liao, Kunyaluk Chaicumpar, Varaporn Vuddhakul, Wieland Meyer, Luciana Trilles, Leo J. J. van Iersel, Jacques F. Meis, Corné H. W. Klaassen, Teun Boekhout

**Affiliations:** 1 Department of Microbiology and Parasitology, Faculty of Medical Sciences, University of Phayao, Phayao, Thailand; 2 CBS-KNAW Fungal Biodiversity Centre, Department of Yeast and Basidiomycete Research, Utrecht, The Netherlands; 3 Department of Internal Medicine and Infectious Diseases, University Medical Center Utrecht, Utrecht University, Utrecht, The Netherlands; 4 Department of Medical Microbiology and Infectious Diseases, Canisius-Wilhelmina Hospital, Nijmegen, The Netherlands; 5 Department of Dermatology, Shanghai Key Laboratory of Molecular Medical Mycology, Institute of Dermatology and Medical Mycology, Changzheng Hospital, Secondary Military Medical University, Shanghai, People's Republic of China; 6 Department of Infectious Disease Epidemiology, Faculty of Medicine, Imperial College London, London, United Kingdom; 7 Division of Mycology, Department of Parasitology, Faculty of Medicine, University of Indonesia, Jakarta, Indonesia; 8 Department of Parasitology, Faculty of Medicine, Christian University of Indonesia, Jakarta, Indonesia; 9 Department of Medical Microbiology, Postgraduate Institute of Medical Education and Research, Chandigarh, India; 10 Department of Medical Mycology, Vallabhbhai Patel Chest Institute, University of Delhi, Delhi, India; 11 Department of Microbiology, Meiji Pharmaceutical University, Tokyo, Japan; 12 Mycology Unit, Microbiology Division, Department of Laboratory Medicine and Pathology, Hamad Medical Corporation, Doha, Qatar; 13 Department of Microbiology, Faculty of Medicine, Health Sciences Centre, Kuwait University, Jabriya, Kuwait; 14 Department of Microbiology, Chinese University of Hong Kong, Hong Kong; 15 Department of Neurology, Faculty of Medicine, University of Indonesia, Jakarta, Indonesia; 16 Department of Neurology, Cipto Mangunkusumo Hospital, Jakarta, Indonesia; 17 Department of Microbiology, Faculty of Medicine, Chiang Mai University, Chiang Mai, Thailand; 18 Research and Diagnostic Center for Emerging Infectious Disease, and Department of Microbiology, Faculty of Medicine, Khon Kaen University, Khon Kaen, Thailand; 19 Department of Microbiology, Faculty of Science, Prince of Songkla University, Hat Yai , Thailand; 20 Molecular Mycology Research Laboratory, Centre for Infectious Diseases and Microbiology, Westmead Millennium Institute, Sydney Medical School–Westmead, The University of Sydney, Westmead Hospital, Sydney, Australia; 21 Laboratório de Micologia, Instituto de Pesquisa Clínica Evandro Chagas, Fundacao Oswaldo Cruz, Rio de Janeiro, Brazil; 22 Centre for Mathematics and Informatics, Amsterdam, The Netherlands; 23 Department of Medical Microbiology, Radboud University Nijmegen Medical Center, Nijmegen, The Netherlands; Instituto de Salud Carlos III, Spain

## Abstract

Cryptococcosis is an important fungal disease in Asia with an estimated 140,000 new infections annually the majority of which occurs in patients suffering from HIV/AIDS. *Cryptococcus neoformans* variety *grubii* (serotype A) is the major causative agent of this disease. In the present study, multilocus sequence typing (MLST) using the ISHAM MLST consensus scheme for the *C*. *neoformans*/*C*. *gattii* species complex was used to analyse nucleotide polymorphisms among 476 isolates of this pathogen obtained from 8 Asian countries. Population genetic analysis showed that the Asian *C*. *neoformans* var. *grubii* population shows limited genetic diversity and demonstrates a largely clonal mode of reproduction when compared with the global MLST dataset. HIV-status, sequence types and geography were found to be confounded. However, a correlation between sequence types and isolates from HIV-negative patients was observed among the Asian isolates. Observations of high gene flow between the Middle Eastern and the Southeastern Asian populations suggest that immigrant workers in the Middle East were originally infected in Southeastern Asia.

## Introduction

Cryptococcosis is one of the main fungal diseases in Asia due to the AIDS pandemic and is caused by members of the *Cryptococcus neoformans*/*C*. *gattii* species complex [1], [2]. In South and Southeast Asia, the number of HIV-infected patients that annually acquire cryptococcosis is estimated to be over 140,000 [3], with the majority of cases being caused by *C. neoformans* var. *grubii* [4]–[12]. The causative agent is an encapsulated opportunistic pathogenic basidiomycetous yeast. Cryptococcosis caused by *C*. *neoformans* var. *grubii* has also been reported to occur in immunocompetent individuals in the Asian region, e.g. from China, Japan, Korea and Taiwan [8], [13]–[16]. In Vietnam, cryptococcosis in both immunocompromised and immunocompetent individuals was found to be mainly caused by *C*. *neoformans* var. *grubii* [17]. *C. neoformans* var. *grubii* (serotype A) has a global distribution and is found in avian excreta, especially from pigeons, and decaying wood [18]–[21]. The other variety, *C*. *neoformans* var. *neoformans* (serotype D), also has a worldwide distribution but is more frequently encountered in Europe [22], [23]. *C. gattii* (serotypes B and C), a sibling species of *C*. *neoformans*, is associated with many tree species in tropical and subtropical regions [18], [24]–[27] and is a major cause of cryptococcal meningitis in immunocompetent individuals. This latter species has also been reported as a causative agent in immunocompromised individuals, particularly HIV-infected patients and solid-organ transplant patients [19], [28]–[31]. Since 1999, *C*. *gattii* emerged in various outbreaks, e.g. at Vancouver Island (British Colombia, Canada), the Pacific Northwest of the United States and more recently in Mediterranean Europe [28], [32]–[35].

Several molecular typing methods, including PCR-fingerprinting, randomly amplified polymorphic DNA (RAPD), PCR-restriction fragment length polymorphism (PCR-RFLP), amplified fragment length polymorphism (AFLP), microsatellite typing, multilocus microsatellite typing (MLMT) and multilocus sequence typing (MLST), have been developed for the investigation of the epidemiology of species belonging to the *C*. *neoformans*/*C. gattii* species complex [30], [31], [36]–[41]. MLST is a typing system that has several advantages over other commonly used typing methods, because the technique is highly reproducible and MLST sequence data can be stored in internet databases, such as http://www.mlst.net/ and http://mlst.mycologylab.org. Thus, the data are portable and exchangeable between laboratories. Recently, seven unlinked genetic loci, i.e. *CAP59*, *GPD1*, IGS1, *LAC1*, *PLB1*, *SOD1* and *URA5*, that represent housekeeping genes, virulence factor coding genes and the intergenic spacer of the ribosomal DNA have been selected for MLST analysis of the *C*. *neoformans*/*C*. *gattii* complex by the International Society of Human and Animal Mycoses (ISHAM) working group on “Genotyping of *C. neoformans* and *C*. *gattii*” [39].

Previous studies that used MLST and AFLP to investigate the population structure of *C*. *neoformans* var. *grubii* showed a correlation between both methods and grouped the isolates into three genetically different subgroups, named AFLP1/VNI, AFLP1A/VNII/VNB and AFLP1B/VNII [36], [39], [42], [43]. The AFLP1/VNI and AFLP1B/VNII genotypes occur globally and form a monophyletic cluster, whereas the AFLP1A/VNB genotype occurs in Southern Africa, especially Botswana, but has also been reported from Brazil [36], [43]. Previously, recombination has been observed within subpopulations in Botswana, but at the global scale reproduction is mainly clonal [43]. MLST has also been used to trace the putative origin of *Cryptococcus* populations [31], [32], [34]. Simwami and coworkers (2011) showed a correlation between MLST types among Thai and African *C*. *neoformans* var. *grubii* isolates that supported the hypothesis of long-distance dispersal from the African continent to Asia within the last 5,000 years [41].

In the current study, MLST was employed to determine the genetic diversity and epidemiological relationships of a collection of clinical and environmental *C*. *neoformans* var. *grubii* isolates that originated from various geographic locations in Asia, including countries from East, South/Southeast Asia and the Middle East. We assessed the extent of recombination that occurs amongst Asian *C*. *neoformans* var. *grubii* isolates. In addition, we determined whether isolates from patients with a different HIV-status could be distinguished using MLST. Finally, we analyzed whether differences in susceptibility to various antifungal drugs correlated with the observed MLST-based genotypic diversity.

## Materials and Methods

### Isolates and media

Three hundred and eleven isolates of *Cryptococcus neoformans* var. *grubii*, including 244 clinical and 67 environmental isolates, were obtained from the following sources: the Chinese *Cryptococcus* Reference Centre at the Second Military Medical University, Shanghai, China (*n*_Clinical_  = 86); the Department of Microbiology, Meiji Pharmaceutical University, Tokyo, Japan (*n*_Clinical_  = 28; *n*_Environmental_  = 10); Prince of Wales Hospital, Hong Kong (*n*_Clinical_  = 14); the Department of Parasitology, Faculty of Medicine, University of Indonesia, Jakarta, Indonesia (*n*_Clinical_  = 40); the Department of Medical Microbiology, Postgraduate Institute of Medical Education and Research, Chandigarh, India (*n*_Clinical_ = 61); the Department of Microbiology, Faculty of Medicine, Health Sciences Centre, Kuwait University, Jabriya, Kuwait (*n*_Clinical_  = 10) and the Mycology Unit, Microbiology Division, Department of Laboratory Medicine and Pathology, Hamad Medical Corporation, Doha, Qatar (*n*_Clinical_  = 5), and the Department of Microbiology, Faculty of Medicine, Chiang Mai University, Chiang Mai, Thailand (*n*_Environmental_  = 57) (Table A and B in Supplementary Tables S1). Furthermore, MLST data obtained from 165 Thai clinical isolates included in a previous study [41] complemented the strain set, resulting in a total of 476 isolates. The MLST profiles were compared to those included in the global MLST dataset (http://mlst.mycologylab.org) and from previous reports [16], [44]. Isolate identification was done as described by Pan *et al*., 2012 [8].

### Mating- and serotype analysis by PCR

Extraction of genomic DNA was performed as previously described [30]. To determine mating- and serotypes, PCR amplifications were applied as described previously [45], [46]. *C. neoformans* strains 125.91 (CBS 10512; aA; AFLP1/VNI), H99 (CBS 8710; αA; AFLP1/VNI), JEC20 (CBS 10511; aD; AFLP2/VNIV), and JEC21 (CBS 10513; αD; AFLP2/VNIV) were used as controls.

### MLST determination

DNA from each isolate was amplified by PCR in 25 µl reaction volumes for each of the seven MLST loci using the primers and protocols described in Table 1. Each amplicon was subsequently sequenced using the BigDye v3.1 Chemistry kit (Applied Biosystems, Foster City, CA) using the same primers as used to obtain the amplicons. Sequencing reaction products were purified with Sephadex G-50 Superfine columns (Amersham Biosciences, Piscataway, NJ) and a MultiScreen HV plate (Millipore, Billerica, MA). An ABI 3700XL DNA analyzer (Applied Biosystems) was used to determine the forward and reverse DNA sequences. Consensus sequences were manually edited using SeqMan v8.0.2 (DNASTAR, Madison, WI) and were subsequently aligned with MEGA v5.05 (www.megasoftware.net). Allele Types (ATs) were assigned to each of the seven loci, resulting in a seven-digit allelic profile for each isolate. The allelic profiles were then defined as Sequence Types (STs) according to the ISHAM MLST consensus scheme for *C*. *neoformans*/*C*. *gattii* species complex (http://mlst.mycologylab.org). All sequences have been deposited in GenBank under the accession number KC529683 to KC533008 (Table C in Supplementary Tables S1) and novel ATs have been added to http://mlst.mycologylab.org/. AT's analysed in Simwami *et al*. (2011) [41] had the indels removed in order to make them compatible for the then-current MLST dataset. However, the current MLST scheme (http://mlst.mycologylab.org) includes indels, and therefore we realigned the entire set of sequences from the latter study in our analyses. This required the reassignment of a number of AT's from the dataset of Simwami *et al*. (2011) [41] (Table A in Supplementary Tables S1). For the global comparison we used data from http://mlst.mycologylab.org and recent reports by Cogliati and colleagues (2013) [44] and Mihara and colleagues (2012) [16].

**Table 1:** Primers used for MLST analysis of Asian *C*. *neoformans* var. *grubii* isolates.

Locus	Primer name	Primer sequence	Amplification conditions	Reference
*CAP59*	CAP59LF	5′ GTGAACAAGCTGCGGC 3′	96°C 5min; 35 cycles: 96°C 30s, 56°C 30s, 72°C 1min;	Hagen et al., 2012b;
	CAP59LR	5′ GGATTCAGTGTGGTGGAAGA 3′	72°C 5min	Fraser et al., 2005
*GPD1*	GPD1LF	5′ GGTTGTCAAGGTTGGAATCAACGG 3′	96°C 5min; 35 cycles: 96°C 30s, 61°C 30s, 72°C 1min;	Hagen et al., 2012b
	GPD1 LR	5′ GGAGCGGAAATGACGACCTTCTT 3′	72°C 5min	
				
IGS1	IGS1F	5′ CAGACGACTTGAATGGGAACG 3′	96°C 5min; 35 cycles: 96°C 30s, 61°C 30s, 72°C 1min;	Bovers et al., 2008b
	IGS2R	5′ ATGCATAGAAAGCTGTTGG 3′	72°C 5min	
*LAC1*	LAC1F	5′ GGCGATACTATTATCGTA 3′	96°C 5min; 35 cycles: 96°C 30s, 52°C 30s, 72°C 1min;	Bovers et al., 2008b
	LAC1R	5′ TTCTGGAGTGGCTAGAGC 3′	72°C 5min	
*PLB1*	PLB1F	5′ CTTCAGGCGGAGAGAGGTTT 3′	96°C 5min; 35 cycles: 96°C 30s, 56°C 30s, 72°C 1min;	Litvintseva et al., 2006
	PLB1R	5′GATTTGGCGTTGGTTTCAGT 3′	72°C 5min	
*SOD1*	JOHE7777	5′ TTCAACCACGAATATGTA 3′	96°C 5min; 35 cycles: 96°C 30s, 52°C 30s, 72°C 1min;	D'Souza et al., 2004
	JOHE7779	5′ AAGCCTCTCATCCATATCTT 3′	72°C 5min	
*URA5*	URA5F	5′ ATGTCCTCCCAAGCCCTCGAC 3′	96°C 5min; 35 cycles: 96°C 30s, 63°C 30s, 72°C 1min;	Meyer et al., 2003
	URA5R	5′ TTAAGACCTCTGAACACCGTACTC 3′	72°C 5min	

### Nucleotide diversity

The determination of the extent of DNA polymorphisms, such as haplotype diversity (*H_d_*), nucleotide diversity (π), number of polymorphic sites (*S*), average number of nucleotide differences (*k*) and Watterson's estimate of the population scaled mutation rate per sequence (θ_s_), were calculated using DnaSP v5.10 (http://www.ub.edu/dnasp/) [47]. Tajima's *D*, Fu & Li's D*, Fu & Li's F* and Fu's *F_s_*, tests for neutrality, were also calculated using DnaSP v5.10. Negative values of these neutrality tests suggest evidence for purifying selection or the excess of high-frequency variants, whereas positive values suggest evidence for balancing or overdominant selection or expansion of rare polymorphisms. Genetic differentiation between populations was estimated using Slatkin linearized *F_ST_* statistics. Estimation of gene flow was assessed using number of migrants per generation (*Nm*).

### Investigation of population structure

The actual number of populations (*K*) among Asian *C*. *neoformans* var. *grubii* in our study was estimated using Structure v2.3.4 (http://pritch.bsd.uchicago.edu/structure.html) [48] and Structure Harvester (http://taylor0.biology.ucla.edu/structureHarvester/) [49]. Twenty-seven simulation runs were conducted for each *K* from 2 to10 using a burn-in of 10^4^ replications and 10^4^ Markov Chain Monte Carlo (MCMC) replications, respectively. The true *K* was calculated from the average and standard deviation of each *K* using the ad hoc statistic implemented in Structure Harvester. Graphic depictions of population genetic structure were drawn from the coefficients of the optimal *K* using CLUMPP v1.1.2 (http://www.stanford.edu/group/rosenberglab/clumpp.html) [50] and Distruct v1.1 (http://www.stanford.edu/group/rosenberglab/distruct.html) [51].

### Recombination

Two common statistics for multilocus linkage disequilibrium analysis, the index of association (*I_A_*) and rBarD, were estimated using Multilocus v1.3b (http://www.agapow.net/software/multilocus/) [52]. These statistics test the null hypothesis of free recombination (i.e. no linkage disequilibrium). The observed values of *I_A_* and rBarD were compared against the expected values obtained with 1,000 randomized data sets. Using these criteria, *p*<0.05 indicates that the null hypothesis of free recombination should be rejected and, consequently, indicates the presence of substantial clonal reproduction. In order to do so, the Pairwise Homoplasy Index (PHI) test implemented in SplitsTree v4.0 (http://www.splitstree.org/) [53] and the pairwise linkage disequilibrium analysis implemented in DnaSP v5.10 using Fisher's exact test were used to detect recombination events among populations using separate alignments for all seven MLST loci. We also used the reticulated network analysis using the CASS algorithm [54] to detect recombination among the Asian population using alignments of concatenated sequences for all seven MLST loci. The genome of *C*. *neoformans* var. *neoformans* strain B3501 ( =  CBS 6900) was used as an outgroup for the CASS network analysis.

### Phylogenetic relationships

The minimum spanning tree that represented the comparison between the original sources of *C*. *neoformans* isolates and their allelic profiles was generated by Phyloviz v1.0 using the goeBURST algorithm (http://goeburst.phyloviz.net/) [55].

Phylogenetic analyses were performed using the Neighbor-joining method with 1,000 bootstrap replicates implemented in MEGA v5.10. The substitution model of this analysis was the uncorrected genetic distances (*p*-distance) model.

### Antifungal susceptibility testing

The susceptibility pattern of seven antifungal drugs, namely amphotericin B (AMB; Bristol Myers Squibb, Woerden, The Netherlands), 5-flucytosine (5FC; Valeant Pharmaceuticals, Zoetermeer, The Netherlands), fluconazole (FLU; Pfizer Central Research, Sandwich, Kent, United Kingdom), itraconazole (ITR; Janssen Cilag, Tilburg, The Netherlands), posaconazole (POS; Schering-Plough Corp., Kenilworth, NJ, USA), voriconazole (VOR; Pfizer Central Research) and isavuconazole (ISA; Basilea Pharmaceutica, Basel, Switzerland) was tested for 14 clinical *C*. *neoformans* var. *grubii* isolates from Hong Kong as described previously [8]. All other data were taken from the Pan *et al.*, 2012 study [8]. Recently published epidemiological cutoff values (ECVs) for *C*. *neoformans* var. *grubii* of AMB, 5FC, FLU, ITR, VOR and POS were implemented in this study. The ECVs for *C*. *neoformans* var. *grubii* of 5FC and FLU is 8 µg/ml, 0.25 µg/ml for ITR, VOR and POS, and 1 µg/ml for amphotericin B, respectively [56], [57].

### Statistical analyses

Analysis of molecular variance (AMOVA), implemented in Arlequin v3.5.1.3, was used to analyze the hierarchical structuring of genetic variation among Asian *C*. *neoformans* populations using the concatenated MLST sequences. Significance was assessed by computing distance pairwise matrices from the MLST sequences using 10,000 permutations [58].

Correlations between sequence types (STs) and origin of *C*. *neoformans* isolates, including geographical origin and HIV-status of patients, and antifungal drug susceptibility profiles (Pan *et al*., 2012) [8] were determined using Chi-square or Fisher's exact tests and binary logistic regression (*p*<0.05). All statistical tests were calculated using GraphPad Prism v5 for Windows (http://www.graphpad.com/prism/prism.htm) (GraphPad Software, San Diego CA) and XLSTAT v2013 (http://www.xlstat.com/) (Addinsoft, NY).

## Results

### MLST analysis

The 476 *C. neoformans* var. *grubii* isolates in our dataset were obtained from 228 HIV-positive patients, 134 HIV-negative patients, and 47 from individuals with unknown HIV status (Table A in Supplementary Tables S1), as well as 67 isolates from avian droppings from Chiang Mai, Thailand and Tokyo, Japan (Table B in Supplementary Tables S1). All isolates possessed mating-type α and serotype A (i.e. were αA). The genetic diversity of the 476 *C. neoformans* var. *grubii* isolates as assessed by MLST revealed 28 sequence types (STs) (Table A, B and D in Supplementary Tables S1), including 4 predominant STs, namely ST4 (*n* = 105; 22.1%), ST5 (*n* = 156; 32.8%), ST6 (*n* = 96; 20.2%), and ST93 (*n* = 52; 10.9%). ST31 and ST77 contained each 14 (2.9%) isolates. The remaining STs were confined to few isolates. Most isolates from the Chinese, Hong Kong, and Japanese populations belonged to ST5. Fourteen percent of the Thai isolates (*n* = 30) belonged to this ST, which was here significantly rarer than in China (Chi-square *p*<0.0001). ST4 and ST6 were found to be the major MLST types in Thailand, while ST93 was dominant in India and Indonesia (Chi-square *p*<0.0001). Fifteen isolates from the Middle East were distributed among 10 STs (Table 2). Most STs from this area consisted of a single isolate, except ST5 and ST31. Most isolates of these latter two STs were obtained from immigrant workers that originated from India, Indonesia, Philippines, and Sudan (Table A in Supplementary Tables S1).

**Table 2:** Distribution of sequence types (STs) of *C*. *neoformans* var. *grubii* isolates among different countries.

Country		Sequence types (STs)																											Total
	4	5	6	23	31	40	53	69	71	77	82	93	141	174	175	176	177	185	186	187	188	189	190	191	192	193	194	195	
**China**	0	**74**	0	0	1	0	5	0	0	0	0	1	0	0	0	0	0	0	1	0	0	0	0	1	0	0	2	1	86
**Hong Kong**	1	**12**	1	0	0	0	0	0	0	0	0	0	0	0	0	0	0	0	0	0	0	0	0	0	0	0	0	0	14
**India**	1	0	2	0	**7**	1	0	0	1	**14**	0	**29**	0	3	0	0	1	0	0	1	0	1	0	0	0	0	0	0	61
**Indonesia**	**8**	0	**9**	0	0	0	0	2	0	0	0	**18**	0	0	0	0	3	0	0	0	0	0	0	0	0	0	0	0	40
**Japan**	0	**36**	0	1	1	0	0	0	0	0	0	0	0	0	0	0	0	0	0	0	0	0	0	0	0	0	0	0	38
**Kuwait**	1	2	0	1	0	0	0	1	0	0	0	1	0	1	1	0	0	1	0	0	0	0	0	0	1	0	0	0	10
**Qatar**	1	2	0	0	2	0	0	0	0	0	0	0	0	0	0	0	0	0	0	0	0	0	0	0	0	0	0	0	5
**Thailand**	**93**	**30**	**84**	0	3	0	1	0	0	0	1	3	1	0	1	1	0	1	0	0	1	0	1	0	0	1	0	0	222
**Total**	105	156	96	2	14	1	6	3	1	14	1	52	1	4	2	1	4	2	1	1	1	1	1	1	1	1	2	1	476

The predominant STs in each country are indicated in bold.

Among the 409 clinical isolates, 24 STs were identified and 16 of them contained clinical isolates only (Tables 3–4). STs 4–6 and 93 were the predominant STs and accounted for 83 (20.3%), 142 (34.7%), 78 (19.1%) and 52 (12.7%) isolates, respectively (Chi-square *p*<0.0001). The remaining STs consisted of few isolates, except ST31 and ST77 that consisted of 10 (2.4%) and 14 (3.4%) isolates, respectively (Table 3). The majority of isolates from HIV-negative patients belonged to ST5 (*n* = 92; 68.7%), while the majority of isolates from HIV-positive people belonged to STs 4, 5, 6 and ST93 that accounted for 72 (31.6%), 27 (11.8%), 68 (29.8%) and 41 (18%) isolates, respectively (Table 3). We investigated how genetic variation was structured across the Asian clinical isolate dataset (i.e. isolates from HIV-positive, -negative and unknown HIV-status patients) using AMOVA. This analysis showed that allelic variation within populations (88.38%) was higher than that observed among populations (11.62%) (*p*<0.0001) (Table 4). When we compared clinical isolates from HIV-positive patients categorized into three regions, East Asia, Middle East and South/Southeast Asia, the variance within populations was approximately 92% (*p* = 0.0654±0.0025), indicating that significant variation in MLST genotypes occurred among individuals within each regional population group. In contrast, MLST genotypic variation within populations of isolates from HIV-negative patients showed less genotypic differences (36.80%) than the variance observed among populations (63.20%) (*p*<0.0001). Chi-squared tests showed a relationship between HIV status and STs (*p*<0.0001; Cramer's V = 0.474) (Sheet S1). A binary logistic regression test showed that ST5 is associated with HIV status (*p*<0.0001) and the standardized (adjusted) Pearson residuals showed that ST5 correlated to isolates obtained from HIV-negative patients (Sheet S1). Of the 92 ST5 isolates, almost all were sampled from East Asia, including China (*n* = 70; 76.1%) and Japan (*n* = 20; 21.7%).

**Table 3:** Distribution of sequence types (STs) of *C*. *neoformans* var. *grubii* isolates according to HIV status of the patients.

HIV status		Sequence types (STs)																											Total
	4	5	6	23	31	40	53	69	71	77	82	93	141	174	175	176	177	185	186	187	188	189	190	191	192	193	194	195	
**HIV-positive**	**72**	**27**	**68**	0	3	0	1	2	1	6	0	**41**	0	1	1	0	3	0	0	0	0	0	0	0	0	0	1	1	228
**HIV-negative**	3	**92**	0	1	7	1	4	1	0	6	0	8	0	3	0	0	1	1	1	1	0	1	0	1	1	0	1	0	134
**Unknown status**	8	23	10	0	0	0	0	0	0	2	1	3	0	0	0	0	0	0	0	0	0	0	0	0	0	0	0	0	47
**Total**	83	142	78	1	10	1	5	3	1	14	1	52	0	4	1	0	4	1	1	1	0	1	0	1	1	0	2	1	409

The predominant STs in each HIV status category are indicated in bold.

**Table 4:** Analysis of Molecular Variance (AMOVA) of Asian *C*. *neoformans* according to HIV status and geographical origin.

	d.f.	Sum of Squares	Variance components (%)	*p*-value
Clinical isolates: HIV-positive (*n* = 227), HIV-negative(*n* = 135), unknown HIV status (*n* = 47)				
Among populations	2	109.66	0.44 (11.62)	
Within populations	406	1364.16	3.36 (88.38)	
Total	408	1473.82	3.80 (100)	0.0000
Isolates from HIV-positive patients : East Asia (*n* = 8), Middle East (*n* = 5), South/Southeast Asia (*n* = 214)				
Among populations	2	14.37	0.30 (7.95)	
Within populations	224	775.92	3.46 (92.05)	
Total	226	790.29	3.76 (100)	0.0654±0.0025
Isolates from HIV-negative patients : East Asia (*n* = 99), Middle East (*n* = 10), South/Southeast Asia (*n* = 26)				
Among populations	2	199.54	3.39 (63.20)	
Within populations	132	260.46	1.97 (36.80)	
Total	134	460.00	5.36 (100)	0.0000

Only clinical and environmental isolates from Chiang Mai, Thailand and Tokyo, Japan could be compared as no environmental isolates could be studied from the other regions. Sixty-seven environmental isolates from Chiang Mai, Thailand, and Tokyo, Japan, belonged to 12 STs from which five STs (i.e. ST141, ST176, ST188, ST190 and ST193) contained environmental isolates only. STs 4, 5 and 6 were the predominant ST types found among the environmental isolates (Chi-square *p*<0.0001). The majority of environmental isolates from Chiang Mai, Thailand, belonged to ST4 (*n* = 22; 38.6%) and 6 (*n* = 18; 31.6%) (Chi-square *p* = 0.0042), while almost all Japanese environmental isolates belonged to ST5 (*n* = 8; 80%) (Fisher's exact test *p* = 0.064) (Table 5).

**Table 5:** Distribution of sequence types (STs) of *C*. *neoformans* isolates from clinical and environmental samples from Thailand and Japan.

Location	Sample		Sequence types (STs)												Total
		4	5	6	23	31	53	141	175	176	185	188	190	193	
**Chiang Mai, Thailand**															
	**Clinical isolates**	**19**	**13**	**11**	0	0	0	0	0	0	0	0	0	0	43
	**Environmental isolates**	**22**	**6**	**18**	0	3	1	1	1	1	1	1	1	1	57
**Tokyo, Japan**															
	**Clinical isolates**	0	**28**	0	0	0	0	0	0	0	0	0	0	0	28
	**Environmental isolates**	0	**8**	0	1	1	0	0	0	0	0	0	0	0	10
**Total**		41	55	29	1	4	1	1	1	1	1	1	1	1	138

The predominant STs in each sample type in these countries are indicated in bold.

### Association between sequence types and geographic origin of Asian C. neoformans isolates

**Figure 1:**
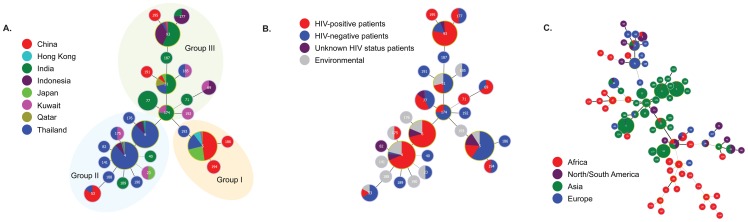
Minimum spanning trees using the goeBURST algorithm showing MLST relationships among Asian C. neoformans var. *grubii* isolates. (A) Tree represents 476 *C*. *neoformans* var. *grubii* isolates from different countries. Each circle represents a unique genotype/sequence type (STs). The size of the circle corresponds to the number of isolates within that genotype. Different colors correspond to different countries; (B) Same as A, but now showing the genotypes from clinical and environmental sources; (C) Same as A and B, but with the addition of the genotypes of 179 *C*. *neoformans* var. *grubii* isolates from different continents (data from http://mlst.mycologylab.org and previous reports by Cogliati *et al*., 2013 [44] and Mihara *et al*., 2012 [16]).

**Figure 2:**
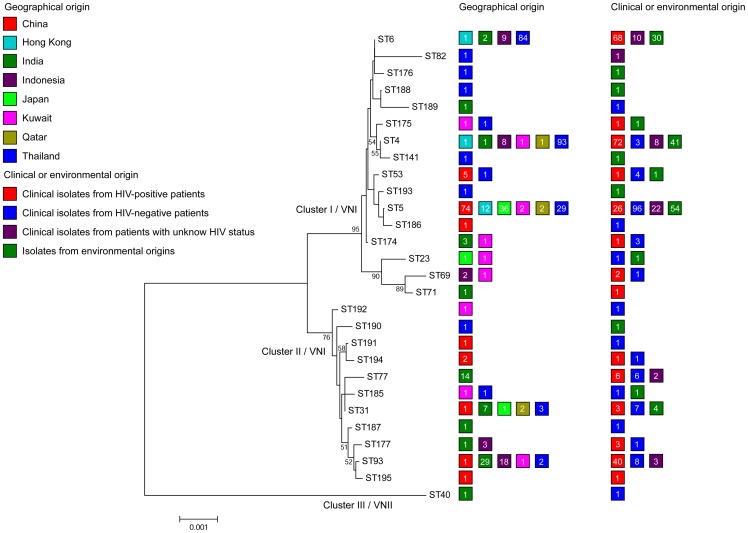
Unrooted Neighbor-joining phylogenetic analysis of the concatenated MLST sequences found among Asian isolates. Numbers at each branch indicate bootstrap values >50%, based on 1,000 replicates. Each color rectangle represents the origin of isolates according to geographic origin, and clinical and environmental origin. Number inside each color rectangle indicates number of isolates.

In order to determine the distribution of STs in different geographical locations, minimum spanning trees and phylogenetic analyses were undertaken based on allelic profiles using the goeBURST algorithm and analysis of concatenated sequences with the Neighbor-joining algorithm, respectively (Figure 1 and 2). Three linages were observed in the minimum spanning tree. Group 1 contained mostly isolates of STs 5, 186, 193 and 194 that originated from China, Hong Kong, and Japan, and also contained 30 out of 222 (13.6%) isolates from Thailand. Group 2 contained mostly isolates from Thailand (*n* = 184; 82.9%). The predominant STs in this group were ST4 and 6. Group 3 comprised most of the Indian and Indonesian isolates that belonged to STs 31, 77 and 93. (Figure 1A).

Phylogenetic analysis of the Asian isolates also showed three clusters. Cluster I/VNI contained three major STs (ST4, 5 and 6) that contained *C*. *neoformans* isolates from China, Hong Kong, Japan, and Thailand. Most Indian and Indonesian isolates occurred in cluster II/VNI, whereas cluster III/VNII contained one ST (ST40) with only isolates from India (Figure 2). The Middle East isolates showed a more scattered distribution (Figure 1A and 2). Two isolates of ST31 came from Qatar, but they were isolated from Indian and Sudanese immigrant workers suggesting that their geographical origins lie elsewhere. Among the clinical isolates, the minimum spanning tree and Maximum Likelihood tree showed an association of the predominant STs, including STs 4, 6 and 93, with *C*. *neoformans* isolates from HIV-positive patients, while ST5, one of the predominant STs, contributed mainly to isolates from HIV-negative patients (Figure 1B and 2; Figure A in Supplementary Figures S1).

**Figure 3:**
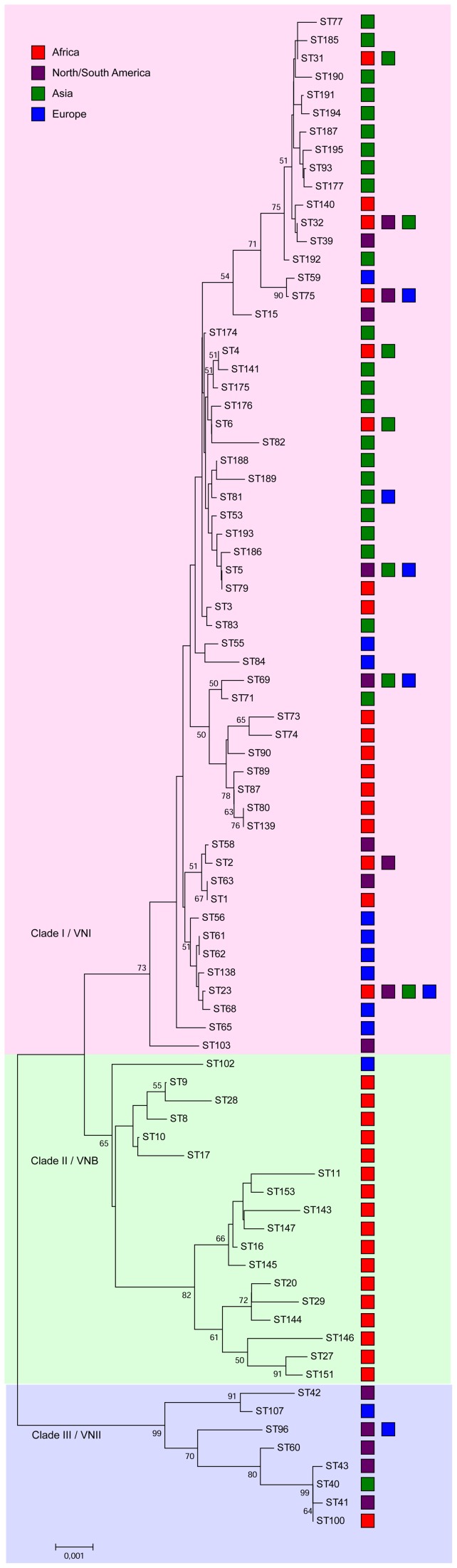
Unrooted Neighbor-joining phylogenetic analysis of the concatenated sequences of each sequence type (ST) found among the global isolates. Numbers at each branch indicate bootstrap values >50%, based on 1,000 replicates. Each color rectangle represents the geographic origin of isolates.

The global *C*. *neoformans* var. *grubii* MLST dataset that contained 179 isolates originating from Africa (*n* = 45), North/South America (*n* = 31), Asia (*n* = 55) and Europe (*n* = 48) was compared using the goeBURST algorithm with the isolates from Asia. Most Asian *C*. *neoformans* isolates clustered together in one group, but a few Asian isolates showed a scattered distribution. Two clusters of African isolates and one cluster of European isolates were observed. Some of isolates from those regions showed a scattered distribution as did the North/South American isolates (Figure 1C). Phylogenetic analysis using Neighbor-joining showed three clades among the global *C*. *neoformans* var. *grubii* isolates. Clade I/VNII contained isolates from Africa, North/South America, Asia and Europe, clade II/VNB contained 17 STs from African isolates and one ST comprising European isolates, and almost all Asian STs occurred in clade I/VNI that also contained isolates from other global regions (*n_Africa_*  = 27, *n_North/South America_*  = 23, *n_Europe_*  = 45) (Figure 3).

### Nucleotide diversity

Nucleotide sequences of all seven loci studied (*CAP59*, *GPD1*, IGS1, *LAC1*, *SOD1*, *PLB1* and *URA5*) had between 6 and 15 polymorphic sites (Table 6). Locus IGS1 had the highest nucleotide diversity (π) of 0.0045, followed by *LAC1* (π = 0.0018) and *GPD1* (π = 0.0014). The average number of nucleotide differences per sequence, i.e. the *k*-value, of most loci ranged from 0.046 to 0.867, except for locus IGS1 that had a higher *k* value of 3.274. Locus *LAC1* showed the highest mutation rate (θ_s_ = 2.226), while the other loci had low θ_s_ values ranging from 0.890 to 2.077. The number of haplotypes (alleles) at each locus ranged from 3 for *CAP59* and *SOD1* to 7 for *LAC1*. Haplotype diversity ranged from 0.013 for *SOD1* to 0.658 for *LAC1*. The neutrality tests, including Tajima's *D*, Fu & Li D*, Fu & Li's F* and Fu's *F_s_* showed significant evidence of purifying selection for all loci, except IGS1 that showed some evidence of balancing selection (Table 6).

**Table 6:** DNA polymorphisms in each MLST locus and concatenated sequences of Asian *C*. *neoformans* var. *grubii* isolates.

Locus	Length	S	γ	θ_s_	*k*	*h*	*H_d_*	*D*	*F_D_*	*F_F_*	*F_s_*
*CAP59*	576	6	0.0001	0.891	0.046	3	0.029	−1.737*	−4.598**	−4.319**	−3.238
*GPD1*	544	11	0.0014	1.633	0.753	6	0.597	−1.173	−3.525**	−3.190**	−0.153
IGS1	725	14	0.0045	2.078	3.274	5	0.327	1.326	0.058	0.669	10.501
*LAC1*	471	15	0.0018	2.226	0.867	7	0.658	−1.427	−6.636**	−5.561**	−0.437
*PLB1*	533	10	0.0005	1.484	0.281	5	0.234	−1.719	−3.840**	−3.686**	−2.192
*SOD1*	536	11	0.0001	1.633	0.051	3	0.013	−2.110**	−6.755**	−6.051**	−3.066
*URA5*	637	11	0.0009	1.633	0.542	5	0.504	−1.455	−5.948**	−5.143**	−0.319
Concatenated	4022	78	0.0015	11.577	5.816	28	0.792	−1.430	−9.170**	−6.446**	−0.520

S: number of polymorphic sites.

π: nucleotide diversity.

θ_s_: Watterson's θ per sequence.

*k*: average number of nucleotide differences per sequence.

h: number of haplotypes.

*H_d_*: haplotype diversity.

*D*, *F_D_*, *F_F_* and *F_s_*: Tajima's D, Fu and Li's D*, Fu and Li's F* and Fu's Fs, respectively. The p value <0.05, *; <0.01, **; <0.001, ***.

**Figure 4:**
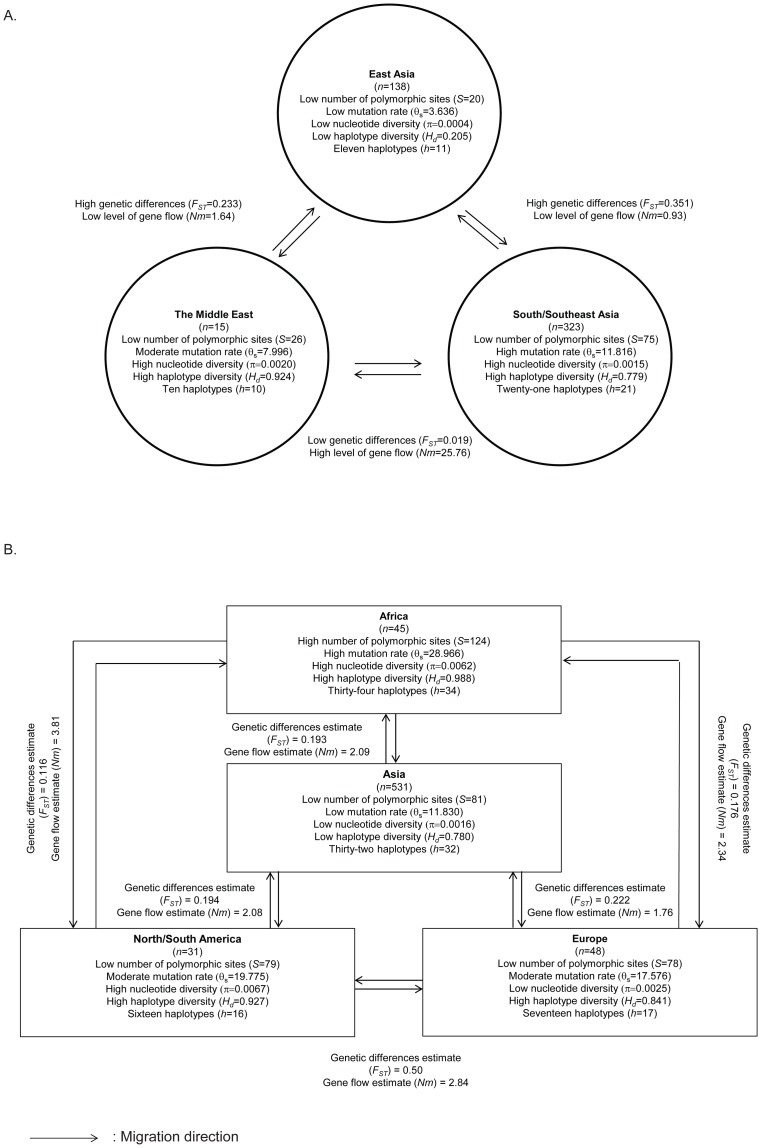
Diagram showing DNA polymorphisms of *C.* *neoformans* var. *grubii* from different Asian regions. (A) DNA polymorphism, genetic differentiation and gene flow; (B) same as A, but comparing African, American, Asian and European populations.

The number of polymorphisms of the concatenated sequences of *C*. *neoformans* var. *grubii* isolates obtained from the East Asian region, including China, Hong Kong and Japan, were lower than those from South/Southeast Asian isolates (i.e. India, Indonesia and Thailand), and those from the Middle East (i.e. Kuwait and Qatar) (Figure 4A and Table E in Supplementary Tables S1). The highest nucleotide diversity (π = 0.002), the highest average number of nucleotide differences per sequence (*k* = 7.962), and the highest haplotype diversity (*H_d_* = 0.924) were found in *C*. *neoformans* isolates from Kuwait and Qatar. *C. neoformans* isolates from South/Southeast Asia had 75 polymorphic site (*S*) and 21 different haplotypes (*h*), and a high mutation rate per sequence (θ_s_ = 11.816). Within the South/Southeast region, haplotype diversity (*H_d_*) of each population was almost similar, while other nucleotide polymorphism estimation values of each population, such as number of polymorphic sites (S), nucleotide diversity (π), mutation rate (θ) and the average number of nucleotide differences per sequence (*k*), were different (Figure 4A, Table E in Supplementary Tables S1). A significant signal of purifying selection was observed in two *C*. *neoformans* populations, namely the one from East Asia (i.e. the Japanese population) and the South/Southeast Asian one (i.e. the Indian population), whereas evidence of a balancing selection or expansion of rare polymorphisms was found in the Indonesian population (Table E in Supplementary Tables S1).

Compared to the global MLST dataset, the Asian population had lower values of nucleotide diversity (π) and haplotype diversity (*H_d_*) of 0.0016 and 0.780, respectively, than those of the African (π = 0.0062; *H_d_* = 0.988), the North/South American (π = 0.0067; *H_d_* = 0.927) and European populations (π = 0.0025; *H_d_* = 0.841) (Figure 4B, Table F in Supplementary Tables S1). The Asian population had lower numbers of polymorphic sites (*S*) and number of haplotypes (*h*) of 81 and 32, respectively, than those from the African populations (*S* = 124; *h* = 34), but they were higher than those from the North/South American (*S* = 79; *h* = 16) and European populations (*S* = 78; *h* = 17) (Figure 4B and Table F in Supplementary Tables S1). Neutrality tests indicated that the variation of all populations was neutral and population sizes did not change. However, the North/South American population showed evidence for a population overdominant selection, whereas the remaining populations showed purifying selection or population expansion.

### Population structure of Asian C. neoformans var. grubii

Genetic differences and the level of gene flow between each of two populations from the three Asian regions studied were estimated using two statistics, *F_ST_* and *Nm*, using concatenated MLST sequences (4,022bp). Genetic differences of the East Asian versus the South/Southeast Asian (*F_ST_*  = 0.351), and the East Asian and Middle East (*F_ST_*  = 0.233) populations were higher than those between the South/Southeast Asian and Middle East populations (*F_ST_*  = 0.019) (Figure 4A, Table G in Supplementary Tables S1). High levels of gene flow, indicated by an *Nm* value of >1, were observed between the South/Southeast Asian population when compared to the Middle East population (*Nm* = 25.76), and, secondly, between the East Asian population and the Middle East population (*Nm* = 1.64). However, the *Nm* value between the South/Southeast Asian and the Middle East populations was much higher than that between the East Asian and the Middle East populations. When the MLST data of the Asian isolates were compared to those from the African, North/South American, and European continents, *F_ST_* and *Nm* estimates were between 0.193 and 0.222, and between 1.76 and 2.09, respectively (Figure 4B, Table H in Supplementary Tables S1), indicating the presence of slight genetic differences, but the occurrence of significant gene flow between the Asian population with those from Africa, North/South America, and Europe.

**Figure 5:**
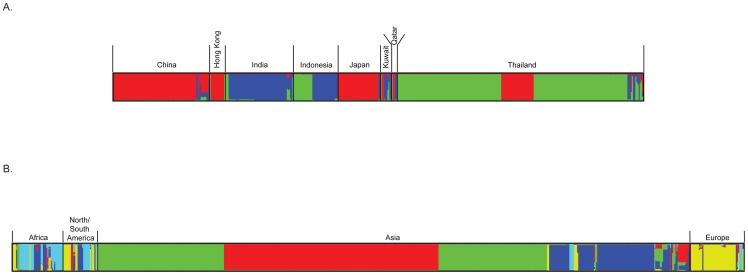
Population structure analysis among Asian *C.* *neoformans* var. *grubii* isolates obtained with the program STRUCTURE. (A) Comparing isolates from different countries and (B) global *C*. *neoformans* var. *grubii* isolates (data from http://mlst.mycologylab.org). The population groups are indicated by different colors. Each bar represents the individual isolates. Mixed color bar represents to share haplotypes in the individual isolate.

Clusters of Asian *C*. *neoformans* var. *grubii* populations were estimated using different numbers of populations that ranged from *K* = 2 to *K* = 10 using Structure. The Evanno method implemented in the Structure Harvester showed the highest delta *K*, an ad hoc statistic, was produced at *K* = 3 (Figure BA in Supplementary Figures S1). This implicates that *K* = 3 seems a good estimate for the actual number of populations included in this study, thus suggesting that three real genetic population clusters occur among the Asian *C*. *neoformans* var. *grubii* isolates that do not fully corroborate the geographically identified populations. The distribution of these three populations differed between the countries (Figure 5A). Almost all cryptococcal isolates from China, Hong Kong and Japan, as well as some isolates from Thailand, belonged to population I. The Thai and part of Indonesian isolates formed population II. Indian isolates, together with part of the Indonesian ones, formed the population III, whereas isolates from Kuwait and Qatar belonged to diverse populations containing genotypes from populations I-III (Figure 5A). Population structure analysis of the global *C*. *neoformans* var. *grubii* isolates showed five genetic populations (*K* = 5) (Figure BB in Supplementary Figures S1). The population structure of the Asian isolates was the same as described above, but two other major populations occurred, namely an African and North/South American population, and an European one. The African and American populations were genetically diverse. Some isolates contained haplotypes occurring among isolates from Asia and Europe and a few of the European isolates shared haplotypes that occurred in isolates from other continents (Figure 5B). However, whether these isolates represent acquisitions from the local environment, or are due to the patient traveling with an *in situ* latent infection is not known and requires further sampling of environmental isolates.

### Recombination

The index of association (*I_A_*) and rBarD values were estimated from the allelic data set to determine the presence of clonality and recombination among Asian *C*. *neoformans* var. *grubii* populations. For all isolates in the entire Asian population and in those from each region (i.e. East Asia, the Middle East and South/Southeast Asia) both *I_A_* and rBarD values strongly rejected the null hypothesis of free recombination (Table 7). However, two recombination events were observed using the pairwise linkage disequilibrium routine implemented in DnaSp (Table I in Supplementary Tables S1). One event occurred among locus *GPD1* and the remaining occurred among locus IGS1. Results of the Pairwise Homoplasy Index (PHI) test (Table J in Supplementary Tables S1) showed that no recombination occurred within each locus across all Asian populations, however two recombination events were observed among concatenated sequences of isolates from East and South/Southeast Asia. CASS analysis (Figure C in Supplementary Figures S1) showed that no recombination occurred among concatenated sequences of Asian isolates. When allelic data that included the global MLST dataset were included, rBarD showed that the overall population genetic structure was in overall significant linkage disequilibrium. The PHI test did not detect recombination events occurring within each locus, but could detect the presence of recombination events among concatenated sequence of the global MLST dataset (Table J in Supplementary Tables S1).

**Table 7:** Linkage disequilibrium analysis amongst Asian *C*. *neoformans* var. *grubii* populations.

		Linkage Disequilibrium			
Population	Sample type	*I_A_*	*p*-value	rBarD	*p*-value
East Asia	all isolates (*n* = 138)	1.750	<0.001	0.397	<0.001
	clone- corrected (*n* = 11)	0.494	0.018	0.100	0.018
Middle East	all isolates (*n* = 15)	0.433	0.043	0.073	0.043
	clone- corrected (*n* = 10)	0.178	0.272	0.030	0.272
South/Southeast Asia	all isolates (*n* = 323)	1.462	<0.001	0.295	<0.001
	clone-corrected (*n* = 22)	0.857	<0.001	0.144	<0.001
Overall	all isolates (*n* = 476)	0.007	<0.001	0.002	<0.001
	clone- corrected (*n* = 28)	0.901	<0.001	0.151	<0.001

*I_A_*: Index of Association.

rBarD: a modified statistics for multilocus linkage disequilibrium analysis.

### *In vitro* antifungal drug susceptibility values

The MIC values of 14 *C. neoformans* var. *grubii* isolates from Hong Kong were determined for seven antifungal drugs, namely AMB, 5FC, FLU, ITR, VOR, POS and ISA (Table 8) and all were susceptible to all antifungal drugs tested. Due to the recent introduction of epidemiological cutoff values, the overall results slightly differ from those presented previously by Pan and colleagues (2012). In this study, 21 clinical isolates (4.4%) from Indonesia (*n* = 13), Thailand (*n* = 5), India (*n* = 2) and China (*n* = 1) showed high MIC values ≥ 16 μg/ml of 5FC. Most of isolates with high 5FC MICs occurred in ST4 (*n* = 3), ST5 (*n* = 2), ST77 (*n* = 2) and ST93 (*n* = 14) (Table A in Supplementary Tables S1). Eight fluconazole (FLU)-resistant isolates (1.7%) occurred in India (*n* = 1), Indonesia (*n* = 5), and Thailand (*n* = 2) and belonged to ST5 (*n* = 1), ST6 (*n* = 1), ST93 (*n* = 5) and ST77 (*n* = 1) (Table A in Supplementary Tables S1). All five 5FC and FLU resistant isolates from Indonesia [8] belonged to ST93. One isolate from Thailand (ST6) showed high MICs for FLU (≥16 μg/ml), but also to VOR (≥0.5 μg/ml) (Table A in Supplementary Tables S1).

**Table 8:** The MIC range, MIC_50_, MIC_90_, and geometric mean for 14 Hong Kong and all 476 *C*. *neoformans* isolates for seven antifungals.

Isolates	Antifungal agent	MIC			
		Range	MIC_50_	Geometric Mean	MIC_90_
Clinical isolates from Hong Kong China (*n* = 14)	Amphotericine B	0.5–1	0.5	0.416	1
	5-Flucytosine	1–8	4	2.895	8
	Fluconazole	0.25–4	2	1.662	4
	Itraconazole	0.031–0.25	0.125	0.086	0.25
	Voriconazole	<0.016–0.125	0.063	0.060	0.125
	Posaconazole	0.031–0.125	0.125	0.083	0.125
	Isavuconazole	<0.016–0.063	0.031	0.028	0.063
All *C*. *neoformans* isolates (*n* = 476)	Amphotericine B	0.063–1	0.25	0.251	0.5
including those from Pan et al., 2012 [8]	5-Flucytosine	<0.063–>64	4	3.483	8
	Fluconazole	0.125–32	2	2.294	4
	Itraconazole	<0.016–0.5	0.063	0.063	0.25
	Voriconazole	<0.016–0.5	0.063	0.049	0.125
	Posaconazole	<0.016–0.25	0.063	0.061	0.125
	Isavuconazole	<0.016–0.125	0.031	0.027	0.063

## Discussion

Previous studies on the genetic structure of *C*. *neoformans* var. *grubii* from Thailand using MLST data showed limited genetic variation [41] with the majority of isolates belonging to STs 4, 5, and 6 (designated as ST44, 45 and 46, respectively, in the original paper by Simwami *et al*., 2011, Table A in Supplementary Tables S1). Two of these predominant STs (i.e. ST4 and ST6) differ only in four nucleotides at a single locus [41]. In the current study, we increased the size of the Asian MLST dataset to include nearly 500 *C*. *neoformans* var. *grubii* isolates originating from three broadly-defined regions, namely East Asia (China, Hong Kong and Japan), South/Southeast Asia (India, Indonesia and Thailand), and the Middle East (Kuwait and Qatar). We found that 99.8% (*n* = 475) of these isolates belonged to lineage VNI, 0.2% (*n* = 1) were VNII and 0% were VNB. The *C. neoformans* var. *grubii* population from the East Asian region showed less genotypic variation than those from South/Southeast Asian and the Middle East regions, and most isolates belonged to ST5. This latter genotype was previously found to be the main ST in China, Japan and South Korea [13], [14], [16] and was reported previously as the MLST M5 genotype [13], [14] (Note that this ST was labelled as ST46 in Simwami *et al*., 2011 [41] [59]). Our data show that ST5 is the major MLST genotype among *C*. *neoformans* var. *grubii* isolates in East Asia. At the global level, and in agreement with previous findings [41], the Asian *C*. *neoformans* var. *grubii* population was found to be less diverse than the African, the North/South American, and the European populations. The population genetic structure of the Asian population was found to be different from the African, and the North/South American and European populations, and contained very few isolates that shared haplotypes occurring in these other populations. On the other hand, the African, North/South American, and European populations also contained some isolates that contained haplotypes occurring among Asian isolates. These findings are in agreement with previous investigations that showed a high genetic diversity of the African population, especially genotype VNB, and less genetic diversity of the Asian population. Note that the VNB lineage also contains also Brazilian isolates [36], [40], [41], [59], [60], thus additional sampling at the global scale may show a broader occurrence of this genotype.

In the current study, no evidence of recombination was detected in the entire Asian region, nor in the smaller regions, or at the global scale using the CASS -, Multilocus (rBarD) -, and PHI (in case of separated loci) analyses. These results suggest that the entire Asian *C*. *neoformans* var. *grubii* population is largely clonal as was previously shown for the Thai population only [41]. However, the pairwise linkage disequilibrium analysis showed a recombination event among sequences of *GPD1* and IGS1 loci and the PHI test also detected recombination events among concatenated sequences in the East and South/Southeast Asia populations. This may be due to non-meiotic reproduction as previously reported [36], [41], [43] amongst isolates that are of the same α-mating type. However, despite this limited recombination, clonal propagation of genotypes predominated leading to a widespread occurrence and overrepresentation of clonal genotypes as has also been seen in several other pathogens, such as *Neisseria meningitides*, *Mycobacterium tuberculosis*, *Fusarium oxysporum*, and *Leishmania tropica* [61].

Our analyses revealed a significant association between predominant sequence types (STs) and their geographical origin in Asia that was not encountered before. These local geographic differences could result from different founder effects and/or regional factors, and may be due to environmental and climate differences [6], [8], [62], [63]. Sequence type ST5 was the predominant MLST genotype found in East Asia and the North of Thailand. Due to the association of *C*. *neoformans* with birds, dispersal may have occurred via the East Asian-Australian flyway [8], [64], and may contribute to the broader distribution of these genotypes. As most bird migrations generally happen twice a year depending on weather conditions, this may also contribute to the observed limited dispersal and low gene flow estimates between East Asia and Northern Thailand. Thus, it seems that Asian *C*. *neoformans* isolates efficiently reproduce clonally, and that rare recombination events may result in an increased genetic variation at some locales, e.g. due to same-sex mating or rare MATα x MATa crossings [65].

A scattered MLST distribution of the Middle East population was observed, similar to previous findings using microsatellite analysis [8]. Owing to a low number of isolates (*n* = 15) with ten haplotypes, the Middle East *C*. *neoformans* var. *grubii* population showed the highest haplotype diversity in our dataset. Seven out of these 15 *C*. *neoformans* var. *grubii* isolates were obtained from immigrant workers, mainly originating from South/Southeast Asia, who may have acquired the yeast in their home countries and, subsequently, carried the pathogen when moving to the Middle East region. Cryptococcal species are known to have a high prevalence of subclinical infection due to infection in childhood [66]. Immigrants and tourists in Europe showed the same phenomenon of being infected by strains that were obtained from their home-country [31], [46], [67]. The observed high level of gene flow between the Middle East and the South/Southeast Asian populations supports this human migration hypothesis. Minimum spanning tree analysis showed that most isolates from India and Indonesia belonged to the same STs (i.e., ST77 and ST93) and a high level of gene flow was observed between these two populations. This observation, unfortunately, is not easy to explain and requires more sampling especially from the environment in these regions.

HIV-status, STs and geographic origins were found to be confounded. However, a significant correlation occurred between the predominant ST5 and HIV-negative patients in Asia. Thus, our study reinforced that genetic differences occur between *C*. *neoformans* var. *grubii* isolates from HIV-positive and HIV-negative patients in Asia [8], [17], [68], [69]. Therefore, this finding may explain the observed correlation of MLST genotypes and HIV status in our study. To unravel the effect of geographically determined genetic variation in the pathogen and its link to HIV status, extensive sampling of environmental isolates is needed across the region in order to decouple the HIV-status of individuals from the geographical origin of the isolates. Next to possible genetically determined differences amongst *Cryptococcus* isolates, human factors, such as anti-interferon-γ and anti-granulocyte-macrophage colony-stimulating factor autoantibodies that have been observed in Taiwanese and Thai patients to be associated with adult-onset immunodeficiency without HIV-infection [70], may contribute to the observed specific pathogen-host correlations.

Recently, epidemiological cutoff values (ECVs) have been defined for the major antifungals against *C*. *neoformans* and *C*. *gattii* [56], [57]. When we used those values, the interpretation of the overall susceptibility results of all isolates, including those presented by Pan *et al*. [8], differed slightly from the interpretation given by these last authors. Twenty-four clinical isolates mainly from Indonesia and Thailand showed high MIC values of 5FC, FLU and VOR. As 5FC is not used in those two countries, resistance to this compound is unlikely to be induced by patient treatment and the origin of this resistance needs further studies.

## Conclusion

MLST typing showed significant genotypic variation between *C*. *neoformans* var. *grubii* populations originating from different Asian regions. Each country had an unique distribution of STs, especially of the predominant STs. Overall, the Asian population showed limited genetic diversity and reproduction is mainly clonal when compared with data from the global *C*. *neoformans* var. *grubii* MLST dataset. A correlation between STs and HIV-negative status, and resistance traits was observed. A largely clonal reproduction strategy helps to maintain these regional differences that are clinically relevant due to their association with the HIV-status of the patients that also differs between the regions studied.

## Supporting Information

Sheet S1 **Chi-square test of Asian C. neoformans according to sequence types (STs) and HIV status.**
(XLS)

Supplementary Figures S1 (DOC)

Supplementary Tables S1 (DOC)
