# Supplementary material for: Geographically Structured Populations of Cryptococcus neoformans Variety grubii in Asia Correlate with HIV Status and Show a Clonal Population Structure
Source: PLoS One. 2013 Sep 3;8(9):e72222. doi: 10.1371/journal.pone.0072222 (PMC3760895; doi:10.1371/journal.pone.0072222)

Figure A. Minimum spanning trees using the goeBURST algorithm showing MLST relationships among 409 clinical Asian *C*. *neoformans* var. *grubii* isolates. Each circle represents a unique genotype/sequence type (STs). The size of the circle corresponds to the number of isolates within that genotype. Different colors correspond to HIV status of patients.


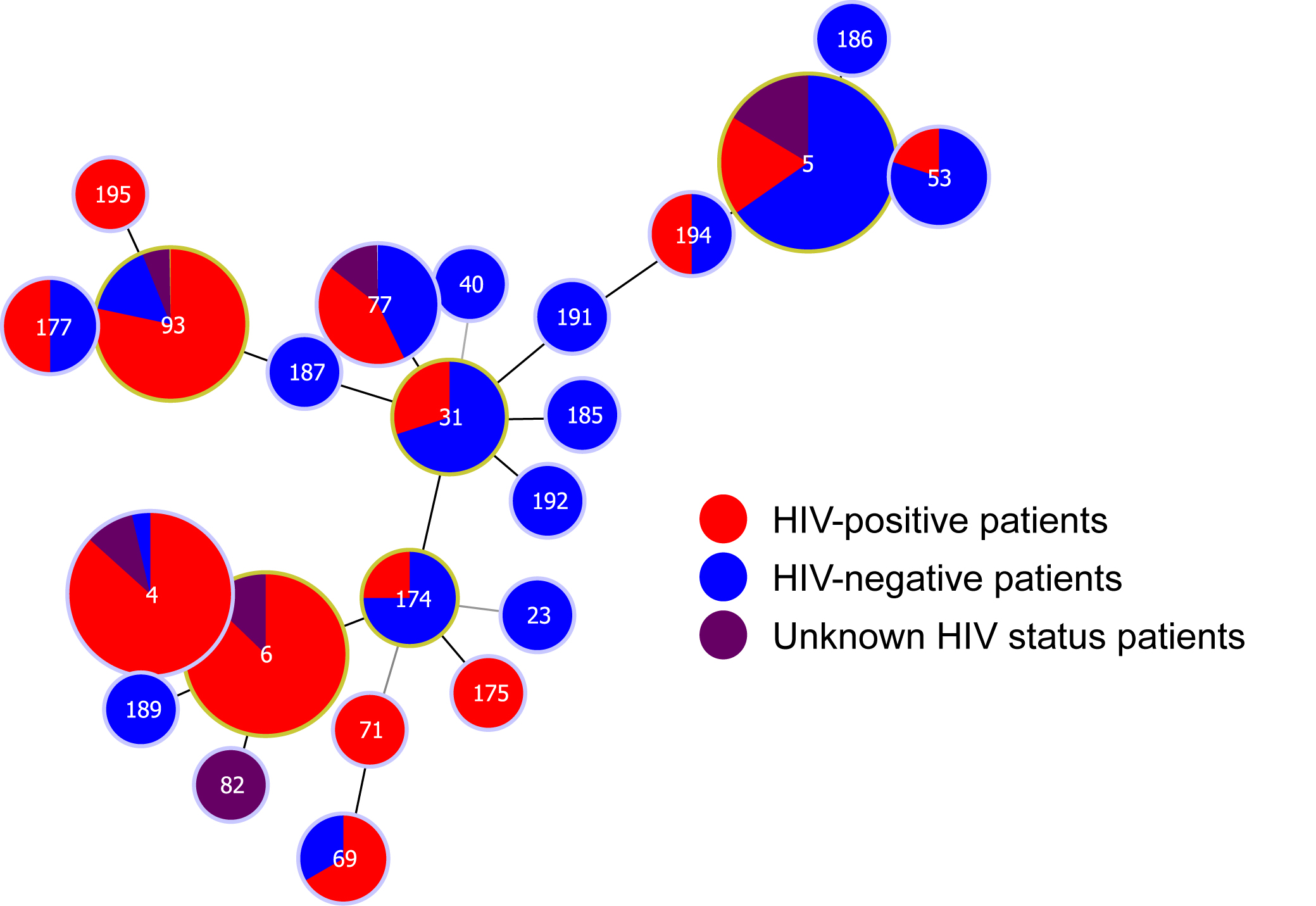


Figure B. Delta *K* values at different *K* values calculating by the Evanno method implemented in the Structure Harvester. A) Delta K values among the Asian *C*. *neoformans* MLST dataset. B) Delta K values among the global *C*. *neoformans* MLST dataset.


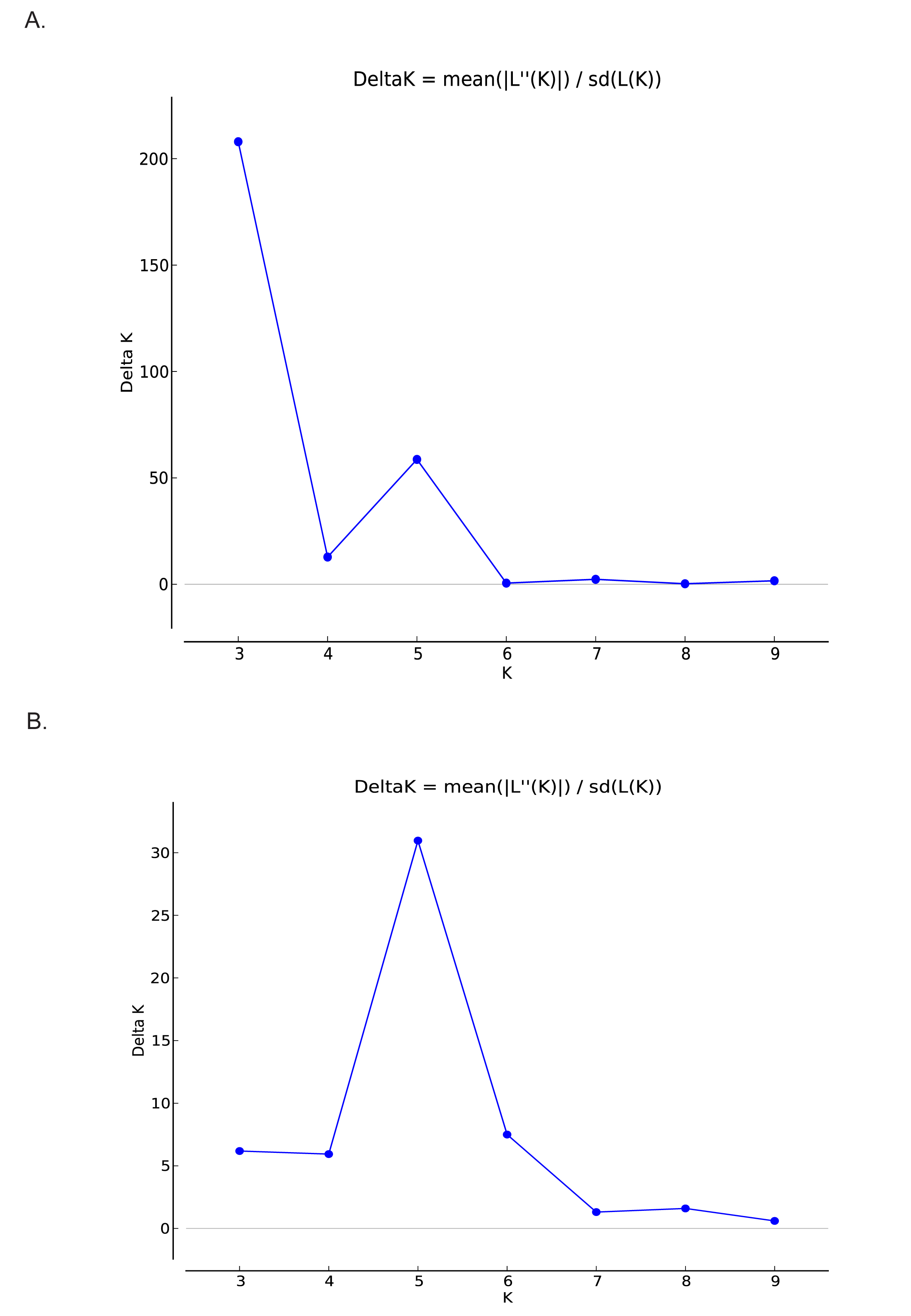


Figure C. A reticulated network analysis using the CASS algorithm of Asian *C*. *neoformans* var. *grubii* for recombination network analysis. No recombination was observed.


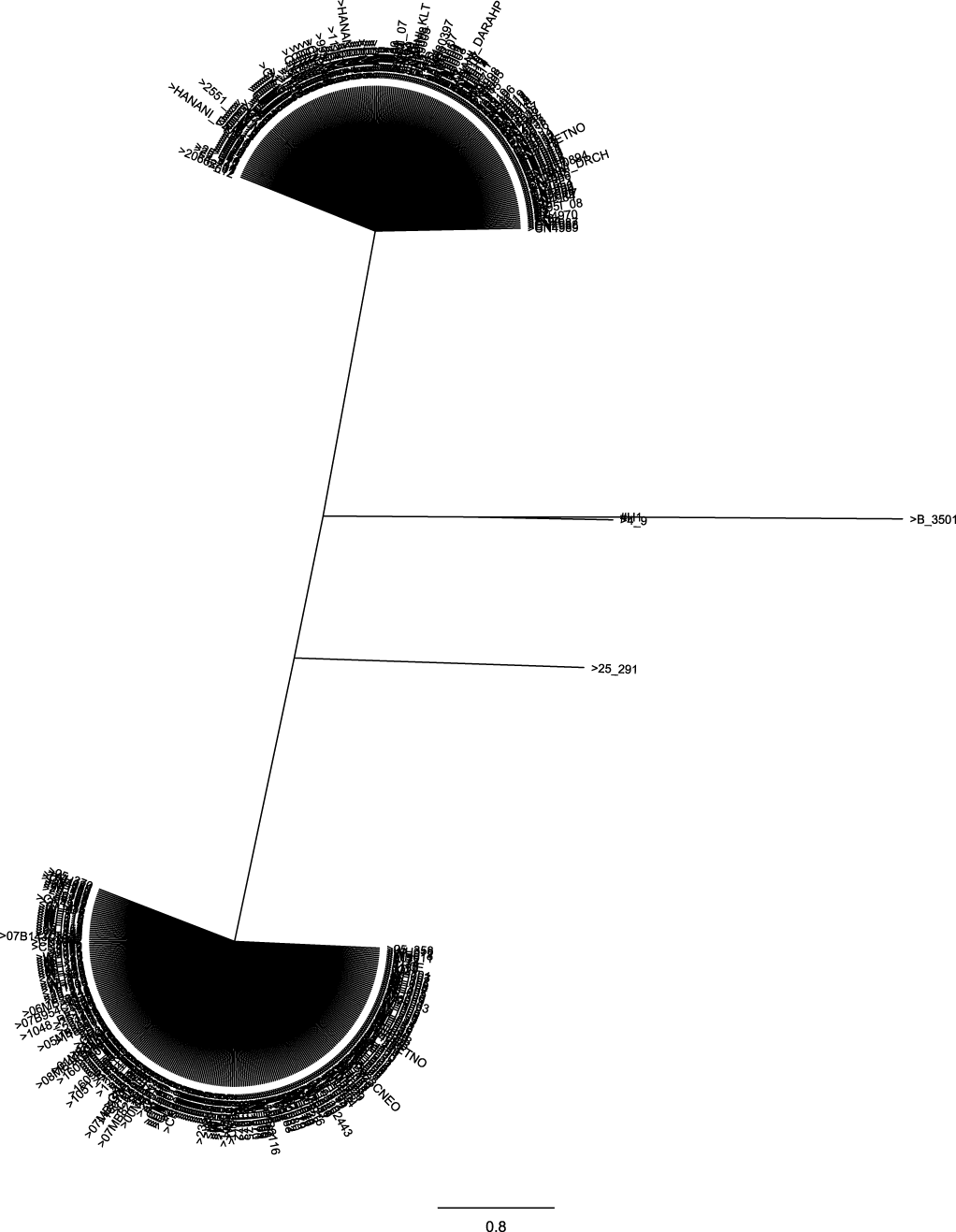

Supplement: Supplementary Figures S1 — (DOC) [file pone.0072222.s002.doc]
