# Supplementary material for: Geographically Structured Populations of Cryptococcus neoformans Variety grubii in Asia Correlate with HIV Status and Show a Clonal Population Structure
Source: PLoS One. 2013 Sep 3;8(9):e72222. doi: 10.1371/journal.pone.0072222 (PMC3760895; doi:10.1371/journal.pone.0072222)
Supplement: Supplementary Tables S1 — (DOC) [file pone.0072222.s003.doc]

Table A. Origin of *Cryptococcus* *neoformans* var. *grubii* MATα, serotype A isolates and clinical background of the patients. The high MIC values isolates are indicated in bold.

| Strain | Sample | Location | Year | HIV status | STs (Sitali et al., 2011) | STs (in this study) |
| --- | --- | --- | --- | --- | --- | --- |
| HK_02 | Clinical | Hong Kong, China | Unknown | Unknown | - | 4 |
| 25_316 | Cerebrospinal fluid | Chandigarh, India | 2007 | Negative | - | 4 |
| 268 | Cerebrospinal fluid | Jakarta, Indonesia | 2006 | Positive | - | 4 |
| 328 | Cerebrospinal fluid | Jakarta, Indonesia | 2006 | Positive | - | 4 |
| 544 | Cerebrospinal fluid | Jakarta, Indonesia | 2006 | Positive | - | 4 |
| 612 | Cerebrospinal fluid | Jakarta, Indonesia | 2007 | Positive | - | 4 |
| 778 | Cerebrospinal fluid | Jakarta, Indonesia | 2007 | Positive | - | 4 |
| 2606 | Cerebrospinal fluid | Jakarta, Indonesia | 2005 | Positive | - | 4 |
| 2597 | Cerebrospinal fluid | Jakarta, Indonesia | 2006 | Positive | - | 4 |
| Jakarta | Cerebrospinal fluid | Jakarta, Indonesia | 2006 | Positive | - | 4 |
| 194_96 | Lymph node biopsy | Kuwait city, Kuwait | 1996 | Positive | - | 4 |
| 1608000894 ^ID^ | Cerebrospinal fluid | Doha, Qatar | 2008 | Positive | - | 4 |
| CN4970 | Cerebrospinal fluid | Chiang Mai, Thailand | 2007 | Positive | 45 | 4 |
| CN4968 | Cerebrospinal fluid | Chiang Mai, Thailand | 2007 | Positive | 45 | 4 |
| CN4955 | Bronchoalveolar lavage | Chiang Mai, Thailand | 2007 | Positive | 45 | 4 |
| CN4938 | Cerebrospinal fluid | Chiang Mai, Thailand | 2006 | Positive | 45 | 4 |
| CN4937 | Cerebrospinal fluid | Chiang Mai, Thailand | 2006 | Positive | 45 | 4 |
| CN4936 | Cerebrospinal fluid | Chiang Mai, Thailand | 2006 | Positive | 45 | 4 |
| CN4934 | Cerebrospinal fluid | Chiang Mai, Thailand | 2006 | Positive | 45 | 4 |
| CN4933 | Cerebrospinal fluid | Chiang Mai, Thailand | 2006 | Positive | 45 | 4 |
| CN4932 | Cerebrospinal fluid | Chiang Mai, Thailand | 2006 | Positive | 45 | 4 |
| CN4931 | Cerebrospinal fluid | Chiang Mai, Thailand | 2006 | Positive | 45 | 4 |
| CN4927 | Cerebrospinal fluid | Chiang Mai, Thailand | 2006 | Positive | 45 | 4 |
| CN4915 | Cerebrospinal fluid | Chiang Mai, Thailand | 2006 | Positive | 45 | 4 |
| CN4914 | Cerebrospinal fluid | Chiang Mai, Thailand | 2006 | Positive | 45 | 4 |
| CN4909 | Cerebrospinal fluid | Chiang Mai, Thailand | 2006 | Positive | 45 | 4 |
| CN4907 | Cerebrospinal fluid | Chiang Mai, Thailand | 2006 | Positive | 45 | 4 |
| CN4905 | Cerebrospinal fluid | Chiang Mai, Thailand | 2006 | Positive | 45 | 4 |
| CN4904 | Cerebrospinal fluid | Chiang Mai, Thailand | 2006 | Positive | 45 | 4 |
| CN4902 | Cerebrospinal fluid | Chiang Mai, Thailand | 2006 | Positive | 45 | 4 |
| CN49008 | Cerebrospinal fluid | Chiang Mai, Thailand | 2007 | Positive | 45 | 4 |
| CN5019 | Blood | Chiang Rai, Thailand | 2008 | Positive | 45 | 4 |
| CN5017 | Cerebrospinal fluid | Chiang Rai, Thailand | 2007 | Positive | 45 | 4 |
| CN5014 | Blood | Chiang Rai, Thailand | 2007 | Positive | 45 | 4 |
| CN5013 | Cerebrospinal fluid | Chiang Rai, Thailand | 2007 | Positive | 45 | 4 |
| CN5011 | Blood | Chiang Rai, Thailand | 2007 | Positive | 45 | 4 |
| CN5009 | Blood | Chiang Rai, Thailand | 2007 | Positive | 45 | 4 |
| CN5005 | Blood | Chiang Rai, Thailand | 2007 | Positive | 45 | 4 |
| CN5003 | Blood | Chiang Rai, Thailand | 2007 | Positive | 45 | 4 |
| CN5002 ^MM^ | Blood | Chiang Rai, Thailand | 2007 | Positive | 45 | 4 |
| CN5001 | Cerebrospinal fluid | Chiang Rai, Thailand | 2007 | Positive | 45 | 4 |
| CN4957 | Cerebrospinal fluid | Chiang Rai, Thailand | Unknown | Positive | 45 | 4 |
| CN4956 | Cerebrospinal fluid | Chiang Rai, Thailand | 2007 | Positive | 45 | 4 |
| 4_319 | Clinical | Khon Kaen, Thailand | Unknown | Unknown | 45 | 4 |
| 11112 | Clinical | Khon Kaen, Thailand | Unknown | Unknown | 45 | 4 |
| 11109 | Clinical | Khon Kaen, Thailand | Unknown | Unknown | 45 | 4 |
| 4_231 | Clinical | Khon Kaen, Thailand | Unknown | Unknown | 45 | 4 |
| **P6*** | Clinical | Khon Kaen, Thailand | Unknown | Unknown | 45 | 4 |
| 4_253 | Clinical | Khon Kaen, Thailand | Unknown | Unknown | 45 | 4 |
| 4_381 | Clinical | Khon Kaen, Thailand | Unknown | Unknown | 45 | 4 |
| CN4954 | Cerebrospinal fluid | Lampang, Thailand | 2007 | Positive | 45 | 4 |
| CN4950 | Cerebrospinal fluid | Lampoon, Thailand | 2007 | Positive | 45 | 4 |
| CN4949 | Cerebrospinal fluid | Lampoon, Thailand | 2007 | Positive | 45 | 4 |
| 50NC2 | Cerebrospinal fluid | Nan, Thailand | 2007 | Positive | 45 | 4 |
| 50NC5 | Cerebrospinal fluid | Nan, Thailand | 2007 | Positive | 45 | 4 |
| 1111I_08 | Blood | Patnai, Thailand | Unknown | Negative | 45 | 4 |
| 2895I_08 | Blood | Patnai, Thailand | Unknown | Negative | 45 | 4 |
| 4500_07 | Blood | Patnai, Thailand | Unknown | Positive | 45 | 4 |
| **20662_07*** | Blood | Songkhla, Thailand | Unknown | Positive | 45 | 4 |
| **28170_07*** | Cerebrospinal fluid | Songkhla, Thailand | Unknown | Positive | 45 | 4 |
| CN4952 | Cerebrospinal fluid | Tak, Thailand | 2007 | Positive | 45 | 4 |
| CM 2 | Cerebrospinal fluid | Ubon Ratchatani, Thailand | 2002 | Positive | 45 | 4 |
| CM 3 | Cerebrospinal fluid | Ubon Ratchatani, Thailand | 2002 | Positive | 45 | 4 |
| CM 4 | Cerebrospinal fluid | Ubon Ratchatani, Thailand | 2002 | Positive | 45 | 4 |
| CM 5 | Cerebrospinal fluid | Ubon Ratchatani, Thailand | 2002 | Positive | 45 | 4 |
| CM 10 | Cerebrospinal fluid | Ubon Ratchatani, Thailand | 2002 | Positive | 45 | 4 |
| CM 14 | Cerebrospinal fluid | Ubon Ratchatani, Thailand | 2002 | Positive | 45 | 4 |
| CM 11 | Cerebrospinal fluid | Ubon Ratchatani, Thailand | 2002 | Positive | 45 | 4 |
| CM 15 | Cerebrospinal fluid | Ubon Ratchatani, Thailand | 2002 | Positive | 45 | 4 |
| CM16 | Cerebrospinal fluid | Ubon Ratchatani, Thailand | 2002 | Positive | 45 | 4 |
| CM 20 | Cerebrospinal fluid | Ubon Ratchatani, Thailand | 2002 | Positive | 45 | 4 |
| CM 24 | Cerebrospinal fluid | Ubon Ratchatani, Thailand | 2002 | Positive | 45 | 4 |
| CM 27 | Cerebrospinal fluid | Ubon Ratchatani, Thailand | 2002 | Positive | 45 | 4 |
| CM 28 | Cerebrospinal fluid | Ubon Ratchatani, Thailand | 2002 | Positive | 45 | 4 |
| CM 29 | Cerebrospinal fluid | Ubon Ratchatani, Thailand | 2002 | Positive | 45 | 4 |
| CM 32 | Cerebrospinal fluid | Ubon Ratchatani, Thailand | 2002 | Positive | 45 | 4 |
| CM 34 | Cerebrospinal fluid | Ubon Ratchatani, Thailand | 2002 | Positive | 45 | 4 |
| CM 36 | Cerebrospinal fluid | Ubon Ratchatani, Thailand | 2002 | Positive | 45 | 4 |
| CM 45 | Cerebrospinal fluid | Ubon Ratchatani, Thailand | 2002 | Positive | 45 | 4 |
| CM 50 | Cerebrospinal fluid | Ubon Ratchatani, Thailand | 2002 | Positive | 45 | 4 |
| CM 52 | Cerebrospinal fluid | Ubon Ratchatani, Thailand | 2002 | Positive | 45 | 4 |
| CM 60 | Cerebrospinal fluid | Ubon Ratchatani, Thailand | 2002 | Positive | 45 | 4 |
| CM 64 | Cerebrospinal fluid | Ubon Ratchatani, Thailand | 2002 | Positive | 45 | 4 |
| WH003 | Sputum | Fujian, China | 1998 | Negative | - | 5 |
| WH004 | Cerebrospinal fluid | Anhui, China | 2001 | Negative | - | 5 |
| WH005 | Cerebrospinal fluid | Jiangsu, China | 2008 | Negative | - | 5 |
| WH006 | Cerebrospinal fluid | Henan, China | 2009 | Negative | - | 5 |
| WH007 | Cerebrospinal fluid | Beijing, China | 2006 | Negative | - | 5 |
| WH008 | Cerebrospinal fluid | Hainan, China | 2009 | Negative | - | 5 |
| WH011 | Cerebrospinal fluid | Anhui, China | 2004 | Negative | - | 5 |
| WH012 | Cerebrospinal fluid | Shanghai, China | 1993 | Negative | - | 5 |
| WH013 | Cerebrospinal fluid | Zhejang, China | 1998 | Negative | - | 5 |
| WH014 | Cerebrospinal fluid | Shandong, China | 2002 | Negative | - | 5 |
| WH015 | Sputum | Guangdong, China | 2003 | Negative | - | 5 |
| WH016 | Cerebrospinal fluid | Guangdong, China | 2002 | Negative | - | 5 |
| WH017 | Cerebrospinal fluid | Guangdong, China | 2004 | Negative | - | 5 |
| WH019 | Cerebrospinal fluid | Guangdong, China | 2003 | Negative | - | 5 |
| WH020 | Blood | Guangdong, China | 2003 | Negative | - | 5 |
| WH022 | Cerebrospinal fluid | Guangdong, China | 2006 | Negative | - | 5 |
| WH023 | Cerebrospinal fluid | Guangdong, China | 2004 | Negative | - | 5 |
| WH025 | Cerebrospinal fluid | Shanghai, China | 2001 | Negative | - | 5 |
| WH026 | Cerebrospinal fluid | Guangdong, China | 2001 | Negative | - | 5 |
| WH027 | Cerebrospinal fluid | Shanghai, China | 2005 | Negative | - | 5 |
| WH028 | Cerebrospinal fluid | Guangdong, China | 2005 | Negative | - | 5 |
| WH030 | Cerebrospinal fluid | Jiangsu, China | 2000 | Negative | - | 5 |
| WH031 | Lung | Shanghai, China | 2000 | Negative | - | 5 |
| WH033 | Skin | Shanghai, China | 2000 | Negative | - | 5 |
| WH034 | Cerebrospinal fluid | Jiangsu, China | 2002 | Negative | - | 5 |
| WH035 | Cerebrospinal fluid | Henan, China | 2007 | Negative | - | 5 |
| WH036 | Cerebrospinal fluid | Shanghai, China | 2003 | Negative | - | 5 |
| WH040 | Cerebrospinal fluid | Shanghai, China | 2003 | Negative | - | 5 |
| WH041 | Cerebrospinal fluid | Guangdong, China | 2006 | Negative | - | 5 |
| WH042 | Cerebrospinal fluid | Shanghai, China | 2004 | Negative | - | 5 |
| WH044 | Cerebrospinal fluid | Beijing, China | 2002 | Negative | - | 5 |
| WH047 | Cerebrospinal fluid | Guangdong, China | 2001 | Negative | - | 5 |
| WH050 | Cerebrospinal fluid | Jiangsu, China | 2007 | Negative | - | 5 |
| WH051 | Cerebrospinal fluid | Guangdong, China | 2005 | Negative | - | 5 |
| WH054 | Cerebrospinal fluid | Guangdong, China | 2004 | Negative | - | 5 |
| WH055 | Cerebrospinal fluid | Hubei, China | 2003 | Negative | - | 5 |
| WH057 | Cerebrospinal fluid | Shanghai, China | 2005 | Negative | - | 5 |
| WH058 | Skin | Guangdong, China | 2004 | Negative | - | 5 |
| WH061 | Cerebrospinal fluid | Guangdong, China | 2007 | Negative | - | 5 |
| **WH062*** | Skin | Hunan, China | 2001 | Negative | - | 5 |
| WH063 | Cerebrospinal fluid | Zhejang, China | 2008 | Negative | - | 5 |
| WH066 | Cerebrospinal fluid | Anhui, China | 2000 | Negative | - | 5 |
| WH067 | Cerebrospinal fluid | Guangdong, China | 2003 | Negative | - | 5 |
| WH068 | Cerebrospinal fluid | Shanghai, China | 1997 | Negative | - | 5 |
| WH072 | Cerebrospinal fluid | Guangdong, China | 2001 | Negative | - | 5 |
| WH075 | Cerebrospinal fluid | Beijing, China | 2006 | Negative | - | 5 |
| WH076 | Cerebrospinal fluid | Guangdong, China | 2005 | Negative | - | 5 |
| WH077 | Cerebrospinal fluid | Shanghai, China | 2001 | Negative | - | 5 |
| WH078 | Cerebrospinal fluid | Hebei, China | 2005 | Negative | - | 5 |
| WH079 | Cerebrospinal fluid | Jiangsu, China | 2005 | Negative | - | 5 |
| WH080 | Cerebrospinal fluid | Jiangsu, China | 2006 | Negative | - | 5 |
| WH082 | Cerebrospinal fluid | Guangdong, China | 2006 | Negative | - | 5 |
| WH083 | Blood | Guangdong, China | 2001 | Negative | - | 5 |
| WH091 | Cerebrospinal fluid | Guangdong, China | 2003 | Negative | - | 5 |
| WH093 | Cerebrospinal fluid | Shandong, China | 1999 | Negative | - | 5 |
| WH094 | Cerebrospinal fluid | Guangdong, China | 2006 | Negative | - | 5 |
| WH096 | Cerebrospinal fluid | Hubei, China | 2003 | Negative | - | 5 |
| WH098 | Cerebrospinal fluid | Shanghai, China | 2006 | Positive | - | 5 |
| WH101 | Cerebrospinal fluid | Zhejang, China | 2007 | Negative | - | 5 |
| WH102 | Cerebrospinal fluid | Shanghai, China | 2002 | Negative | - | 5 |
| WH104 | Cerebrospinal fluid | Zhejang, China | 1996 | Negative | - | 5 |
| WH105 | Cerebrospinal fluid | Shanghai, China | 2004 | Negative | - | 5 |
| WH106 | Cerebrospinal fluid | Jiangsu, China | 1998 | Negative | - | 5 |
| WH108 | Cerebrospinal fluid | Shanghai, China | 2005 | Negative | - | 5 |
| WH114 | Cerebrospinal fluid | Zhejang, China | 2000 | Negative | - | 5 |
| WH115 | Cerebrospinal fluid | Jiangsu, China | 1994 | Negative | - | 5 |
| WH117 | Cerebrospinal fluid | Hebei, China | 1999 | Negative | - | 5 |
| WH118 | Cerebrospinal fluid | Shanghai, China | 1995 | Negative | - | 5 |
| WH119 | Cerebrospinal fluid | Zhejang, China | 2001 | Negative | - | 5 |
| WH120 | Cerebrospinal fluid | Sichuan, China | 2003 | Negative | - | 5 |
| WH121 | Blood | Guangdong, China | 2007 | Positive | - | 5 |
| WH122 | Cerebrospinal fluid | Guangdong, China | 2006 | Positive | - | 5 |
| WH124 | Cerebrospinal fluid | Guangdong, China | 2007 | Positive | - | 5 |
| WH126 | Cerebrospinal fluid | Guangdong, China | 2007 | Positive | - | 5 |
| HK_03 | Clinical | Hong Kong, China | Unknown | Unknown | - | 5 |
| HK_04 | Clinical | Hong Kong, China | Unknown | Unknown | - | 5 |
| HK_05 | Clinical | Hong Kong, China | Unknown | Unknown | - | 5 |
| HK_06 | Clinical | Hong Kong, China | Unknown | Unknown | - | 5 |
| HK_07 | Clinical | Hong Kong, China | Unknown | Unknown | - | 5 |
| HK_08 | Clinical | Hong Kong, China | Unknown | Unknown | - | 5 |
| HK_09 | Clinical | Hong Kong, China | Unknown | Unknown | - | 5 |
| HK_10 | Clinical | Hong Kong, China | Unknown | Unknown | - | 5 |
| HK_11 | Clinical | Hong Kong, China | Unknown | Unknown | - | 5 |
| HK_12 | Clinical | Hong Kong, China | Unknown | Unknown | - | 5 |
| HK_13 | Clinical | Hong Kong, China | Unknown | Unknown | - | 5 |
| HK_14 | Clinical | Hong Kong, China | Unknown | Unknown | - | 5 |
| 9104 | Skin | Tokyo, Japan | 1988 | Negative | - | 5 |
| 9106 | Cerebrospinal fluid | Tokyo, Japan | 1988 | Negative | - | 5 |
| 9107 | Unknown | Tokyo, Japan | 1988 | Negative | - | 5 |
| 9108 | Skin | Tokyo, Japan | 1987 | Negative | - | 5 |
| 9111 | Cerebrospinal fluid | Tokyo, Japan | 1986 | Negative | - | 5 |
| 9114 | Unknown | Tokyo, Japan | Unknown | Negative | - | 5 |
| 9165 | Cerebrospinal fluid | Tokyo, Japan | 1983 | Negative | - | 5 |
| 9166 | Skin | Tokyo, Japan | 1983 | Negative | - | 5 |
| 9167 | Cerebrospinal fluid | Tokyo, Japan | 1983 | Negative | - | 5 |
| 9170 | Skin | Tokyo, Japan | 1984 | Negative | - | 5 |
| 9172 | Cerebrospinal fluid | Tokyo, Japan | 1984 | Unknown | - | 5 |
| 9173 | Cerebrospinal fluid | Tokyo, Japan | 1985 | Negative | - | 5 |
| 9174 | Cerebrospinal fluid | Tokyo, Japan | 1985 | Negative | - | 5 |
| 9179 | Unknown | Tokyo, Japan | 1987 | Negative | - | 5 |
| 9197 | Unknown | Tokyo, Japan | 1989 | Unknown | - | 5 |
| 9198 | Unknown | Tokyo, Japan | 1989 | Unknown | - | 5 |
| 9199 | Unknown | Tokyo, Japan | 1989 | Unknown | - | 5 |
| 9204 | Unknown | Tokyo, Japan | 1989 | Unknown | - | 5 |
| 9205 | Unknown | Tokyo, Japan | 1989 | Unknown | - | 5 |
| 9213 | Unknown | Tokyo, Japan | 1989 | Unknown | - | 5 |
| 9217 | Unknown | Tokyo, Japan | 1995 | Unknown | - | 5 |
| 9237 | Skin | Tokyo, Japan | 1994 | Negative | - | 5 |
| 9238 | Cerebrospinal fluid | Tokyo, Japan | 1990 | Negative | - | 5 |
| 9239 | Skin | Tokyo, Japan | 1990 | Negative | - | 5 |
| 9251 | Skin | Tokyo, Japan | 2001 | Negative | - | 5 |
| 9263 | Skin | Tokyo, Japan | 2003 | Negative | - | 5 |
| 9264 | Skin | Tokyo, Japan | 2002 | Negative | - | 5 |
| 9265 | Skin | Tokyo, Japan | 2001 | Negative | - | 5 |
| 1185_04 ^IN^ | Endotracheal secretion | Kuwait city, Kuwait | 2004 | Negative | - | 5 |
| 201_95 | Lumbar swelling aspirate | Kuwait city, Kuwait | 1995 | Negative | - | 5 |
| 1607001262 ^ID^ | Cerebrospinal fluid | Doha, Qatar | 2008 | Positive | - | 5 |
| 1609290340 ^PH^ | Cerebrospinal fluid | Doha, Qatar | 2009 | Negative | - | 5 |
| CM30 | Cerebrospinal fluid | Ubon Ratchatani, Thailand | 2002 | Positive | 46 | 5 |
| **CN48*** | Clinical | Khon Kaen, Thailand | Unknown | Unknown | 46 | 5 |
| CN49004 | Cerebrospinal fluid | Chiang Mai, Thailand | 2007 | Positive | 46 | 5 |
| CN49006 | Cerebrospinal fluid | Chiang Mai, Thailand | 2007 | Positive | 46 | 5 |
| CN4906 | Cerebrospinal fluid | Chiang Mai, Thailand | 2006 | Positive | 46 | 5 |
| CN4916 | Cerebrospinal fluid | Chiang Mai, Thailand | 2006 | Positive | 46 | 5 |
| CN4920 | Cerebrospinal fluid | Chiang Mai, Thailand | 2006 | Positive | 46 | 5 |
| CN4921 | Cerebrospinal fluid | Mae Hong Son, Thailand | 2006 | Positive | 46 | 5 |
| **CN4924^#^** | Cerebrospinal fluid | Chiang Mai, Thailand | 2006 | Positive | 46 | 5 |
| CN4946 | Cerebrospinal fluid | Chiang Mai, Thailand | 2006 | Positive | 46 | 5 |
| CN4948 | Cerebrospinal fluid | Chiang Mai, Thailand | 2007 | Positive | 46 | 5 |
| CN4960 | Cerebrospinal fluid | Chiang Rai, Thailand | 2007 | Positive | 46 | 5 |
| CN4967 | Cerebrospinal fluid | Chiang Mai, Thailand | 2007 | Positive | 46 | 5 |
| CN4977 | Cerebrospinal fluid | Chiang Mai, Thailand | 2007 | Positive | 46 | 5 |
| CN4980 | Cerebrospinal fluid | Chiang Mai, Thailand | 2007 | Positive | 46 | 5 |
| CN4983 | Cerebrospinal fluid | Chiang Mai, Thailand | 2007 | Positive | 46 | 5 |
| CN4993 | Cerebrospinal fluid | Chiang Mai, Thailand | 2007 | Positive | 46 | 5 |
| CN5008 | Cerebrospinal fluid | Chiang Rai, Thailand | 2007 | Positive | 46 | 5 |
| CN5012 | Cerebrospinal fluid | Chiang Rai, Thailand | 2007 | Positive | 46 | 5 |
| CN5015 | Cerebrospinal fluid | Chiang Rai, Thailand | 2007 | Positive | 46 | 5 |
| CN5018 | Blood | Chiang Rai, Thailand | 2008 | Positive | 46 | 5 |
| 1-488 | Clincal | Khon Kaen, Thailand | Unknown | Unknown | 46 | 5 |
| 1-489 | Clinical | Khon Kaen, Thailand | Unknown | Unknown | 46 | 5 |
| CM 30 | Cerebrospinal fluid | Ubon Ratchatani, Thailand | 2002 | Positive | 46 | 5 |
| HK_01 | Clinical | Hong Kong, China | Unknown | Unknown | - | 6 |
| 25_372 | Cerebrospinal fluid | Assam, India | 2009 | Positive | - | 6 |
| 25_373 | Cerebrospinal fluid | Chandigarh, India | 2009 | Positive | - | 6 |
| 267 | Cerebrospinal fluid | Jakarta, Indonesia | 2006 | Positive | - | 6 |
| 2339 | Cerebrospinal fluid | Jakarta, Indonesia | 2006 | Positive | - | 6 |
| 2594 | Cerebrospinal fluid | Jakarta, Indonesia | 2006 | Positive | - | 6 |
| 3187 | Cerebrospinal fluid | Jakarta, Indonesia | 2006 | Positive | - | 6 |
| Jakarta (P) | Blood | Jakarta, Indonesia | 2006 | Positive | - | 6 |
| Jakarta (H) | Blood | Jakarta, Indonesia | 2006 | Positive | - | 6 |
| Jakarta (KT) | Skin | Jakarta, Indonesia | 2006 | Positive | - | 6 |
| Jakarta KLT | Skin | Jakarta, Indonesia | 2006 | Positive | - | 6 |
| Jakarta RTL | Skin | Jakarta, Indonesia | 2006 | Positive | - | 6 |
| CN5010 | Blood | Chiang Rai, Thailand | 2007 | Positive | 44 | 6 |
| CN4998 | Cerebrospinal fluid | Chiang Mai, Thailand | 2007 | Positive | 44 | 6 |
| CN4995 | Cerebrospinal fluid | Chiang Mai, Thailand | 2007 | Positive | 44 | 6 |
| CN4989 | Cerebrospinal fluid | Chiang Mai, Thailand | 2007 | Positive | 44 | 6 |
| CN4988 | Cerebrospinal fluid | Chiang Mai, Thailand | 2007 | Positive | 44 | 6 |
| CN4987 | Cerebrospinal fluid | Chiang Mai, Thailand | 2007 | Positive | 44 | 6 |
| **CN4964^#,¶^** | Cerebrospinal fluid | Chiang Mai, Thailand | 2007 | Positive | 44 | 6 |
| CN4947 | Cerebrospinal fluid | Chiang Rai, Thailand | 2006 | Positive | 44 | 6 |
| CN4945 | Cerebrospinal fluid | Chiang Rai, Thailand | 2006 | Positive | 44 | 6 |
| CN4944 | Cerebrospinal fluid | Chiang Mai, Thailand | 2006 | Positive | 44 | 6 |
| CN4943 | Cerebrospinal fluid | Chiang Rai, Thailand | 2006 | Positive | 44 | 6 |
| CN4942 | Cerebrospinal fluid | Lampang, Thailand | 2006 | Positive | 44 | 6 |
| CN4941 | Cerebrospinal fluid | Chiang Mai, Thailand | 2006 | Positive | 44 | 6 |
| CN4940 | Cerebrospinal fluid | Chiang Mai, Thailand | 2006 | Positive | 44 | 6 |
| CN4926 | Cerebrospinal fluid | Chiang Rai, Thailand | 2006 | Positive | 44 | 6 |
| CN4919 | Cerebrospinal fluid | Chiang Rai, Thailand | 2006 | Positive | 44 | 6 |
| CN4918 | Cerebrospinal fluid | Chiang Rai, Thailand | 2006 | Positive | 44 | 6 |
| CN4917 | Cerebrospinal fluid | Chiang Rai, Thailand | 2006 | Positive | 44 | 6 |
| CN4903 | Cerebrospinal fluid | Chiang Rai, Thailand | 2006 | Positive | 44 | 6 |
| CN4901 | Cerebrospinal fluid | Chiang Mai, Thailand | 2006 | Positive | 44 | 6 |
| CN49005 | Cerebrospinal fluid | Chiang Mai, Thailand | 2007 | Positive | 44 | 6 |
| 4-187 | Clinical | Khon Kaen, Thailand | Unknown | Unknown | 44 | 6 |
| 269 | Clinical | Khon Kaen, Thailand | Unknown | Unknown | 44 | 6 |
| 4-315 | Clinical | Khon Kaen, Thailand | Unknown | Unknown | 44 | 6 |
| 1-587 | Clinical | Khon Kaen, Thailand | Unknown | Unknown | 44 | 6 |
| 1219 | Clinical | Khon Kaen, Thailand | Unknown | Unknown | 44 | 6 |
| 4_83 | Clinical | Khon Kaen, Thailand | Unknown | Unknown | 44 | 6 |
| 1-588 | Clinical | Khon Kaen, Thailand | Unknown | Unknown | 44 | 6 |
| 4-202 | Clinical | Khon Kaen, Thailand | Unknown | Unknown | 44 | 6 |
| 1-846 | Clinical | Khon Kaen, Thailand | Unknown | Unknown | 44 | 6 |
| 2551-07 | Cerebrospinal fluid | Songkhla, Thailand | Unknown | Positive | 44 | 6 |
| 2550 II-07 | Blood | Songkhla, Thailand | Unknown | Positive | 44 | 6 |
| 2461_07 | Cerebrospinal fluid | Songkhla, Thailand | Unknown | Positive | 44 | 6 |
| CM 1 | Cerebrospinal fluid | Ubon Ratchatani, Thailand | 2002 | Positive | 44 | 6 |
| CM 6 | Cerebrospinal fluid | Ubon Ratchatani, Thailand | 2002 | Positive | 44 | 6 |
| CM 7 | Cerebrospinal fluid | Ubon Ratchatani, Thailand | 2002 | Positive | 44 | 6 |
| CM 8 | Cerebrospinal fluid | Ubon Ratchatani, Thailand | 2002 | Positive | 44 | 6 |
| CM 12 | Cerebrospinal fluid | Ubon Ratchatani, Thailand | 2002 | Positive | 44 | 6 |
| CM 13 | Cerebrospinal fluid | Ubon Ratchatani, Thailand | 2002 | Positive | 44 | 6 |
| CM 17 | Cerebrospinal fluid | Ubon Ratchatani, Thailand | 2002 | Positive | 44 | 6 |
| CM 18 | Cerebrospinal fluid | Ubon Ratchatani, Thailand | 2002 | Positive | 44 | 6 |
| CM 22 | Cerebrospinal fluid | Ubon Ratchatani, Thailand | 2002 | Positive | 44 | 6 |
| CM 23 | Cerebrospinal fluid | Ubon Ratchatani, Thailand | 2002 | Positive | 44 | 6 |
| CM 25 | Cerebrospinal fluid | Ubon Ratchatani, Thailand | 2002 | Positive | 44 | 6 |
| CM 26 | Cerebrospinal fluid | Ubon Ratchatani, Thailand | 2002 | Positive | 44 | 6 |
| CM 33 | Cerebrospinal fluid | Ubon Ratchatani, Thailand | 2002 | Positive | 44 | 6 |
| CM 37 | Cerebrospinal fluid | Ubon Ratchatani, Thailand | 2002 | Positive | 44 | 6 |
| CM 38 | Cerebrospinal fluid | Ubon Ratchatani, Thailand | 2002 | Positive | 44 | 6 |
| CM 39 | Cerebrospinal fluid | Ubon Ratchatani, Thailand | 2002 | Positive | 44 | 6 |
| CM 40 | Cerebrospinal fluid | Ubon Ratchatani, Thailand | 2002 | Positive | 44 | 6 |
| CM 41 | Cerebrospinal fluid | Ubon Ratchatani, Thailand | 2002 | Positive | 44 | 6 |
| CM42 | Cerebrospinal fluid | Ubon Ratchatani, Thailand | 2002 | Positive | 44 | 6 |
| CM 43 | Cerebrospinal fluid | Ubon Ratchatani, Thailand | 2002 | Positive | 44 | 6 |
| CM 44 | Cerebrospinal fluid | Ubon Ratchatani, Thailand | 2002 | Positive | 44 | 6 |
| CM 46 | Cerebrospinal fluid | Ubon Ratchatani, Thailand | 2002 | Positive | 44 | 6 |
| CM 47 | Cerebrospinal fluid | Ubon Ratchatani, Thailand | 2002 | Positive | 44 | 6 |
| CM 48 | Cerebrospinal fluid | Ubon Ratchatani, Thailand | 2002 | Positive | 44 | 6 |
| CM 49 | Cerebrospinal fluid | Ubon Ratchatani, Thailand | 2002 | Positive | 44 | 6 |
| CM 51 | Cerebrospinal fluid | Ubon Ratchatani, Thailand | 2002 | Positive | 44 | 6 |
| CM 55 | Cerebrospinal fluid | Ubon Ratchatani, Thailand | 2002 | Positive | 44 | 6 |
| CM 56 | Cerebrospinal fluid | Ubon Ratchatani, Thailand | 2002 | Positive | 44 | 6 |
| CM 57 | Cerebrospinal fluid | Ubon Ratchatani, Thailand | 2002 | Positive | 44 | 6 |
| CM 58 | Cerebrospinal fluid | Ubon Ratchatani, Thailand | 2002 | Positive | 44 | 6 |
| CM 59 | Cerebrospinal fluid | Ubon Ratchatani, Thailand | 2002 | Positive | 44 | 6 |
| CM 61 | Cerebrospinal fluid | Ubon Ratchatani, Thailand | 2002 | Positive | 44 | 6 |
| CM 63 | Cerebrospinal fluid | Ubon Ratchatani, Thailand | 2002 | Positive | 44 | 6 |
| 8_92 | Cerebrospinal fluid | Kuwait city, Kuwait | Unknown | Negative | - | 23 |
| WH037 | Cerebrospinal fluid | Henan, China | 2003 | Negative | - | 31 |
| 25_18 | Cerebrospinal fluid | Chandigarh, India | 1989 | Negative | - | 31 |
| 25_229 | Cerebrospinal fluid | Chandigarh, India | 2005 | Positive | - | 31 |
| 25_266 | Cerebrospinal fluid | Chandigarh, India | 2006 | Negative | - | 31 |
| 25_298 | Cerebrospinal fluid | Chandigarh, India | 2007 | Negative | - | 31 |
| 25_355 | Cerebrospinal fluid | Chandigarh, India | 2009 | Negative | - | 31 |
| 25_357 | Cerebrospinal fluid | Chandigarh, India | 2009 | Positive | - | 31 |
| 25_370 | Cerebrospinal fluid | Assam, India | 2009 | Positive | - | 31 |
| 1608000352 ^SD^ | Cerebrospinal fluid | Doha, Qatar | 2008 | Negative | - | 31 |
| 1605202443 ^IN^ | Cerebrospinal fluid | Doha, Qatar | 2005 | Negative | - | 31 |
| WH018 | Cerebrospinal fluid | Guangdong, China | 2003 | Negative | - | 53 |
| WH070 | Cerebrospinal fluid | Shanghai, China | 1993 | Negative | - | 53 |
| WH073 | Cerebrospinal fluid | Shanghai, China | 1999 | Negative | - | 53 |
| WH113 | Cerebrospinal fluid | Anhui, China | 2007 | Negative | - | 53 |
| WH125 | Cerebrospinal fluid | Guangdong, China | 2007 | Positive | - | 53 |
| 25_291 | Cerebrospinal fluid | Chandigarh, India | 2006 | Negative | - | 40 |
| 2478 | Cerebrospinal fluid | Jakarta, Indonesia | 2006 | Positive | - | 69 |
| 264 | Cerebrospinal fluid | Jakarta, Indonesia | 2006 | Positive | - | 69 |
| 110_99 | Blood | Kuwait city, Kuwait | 1999 | Negative | - | 69 |
| 25_328 | Cerebrospinal fluid | Chandigarh, India | 2007 | Positive | - | 71 |
| 25_104 | Cerebrospinal fluid | Chandigarh, India | 1999 | Positive | - | 77 |
| 25_105 | Cerebrospinal fluid | Chandigarh, India | 2000 | Negative | - | 77 |
| 25_110 | Cerebrospinal fluid | Chandigarh, India | 1987 | Negative | - | 77 |
| 25_14 | Clinical | India | Unknown | Unknown | - | 77 |
| **25_244*** | Cerebrospinal fluid | Chandigarh, India | 2005 | Positive | - | 77 |
| 25_365 | Cerebrospinal fluid | Chandigarh, India | 2009 | Positive | - | 77 |
| 25_369 | Cerebrospinal fluid | Assam, India | 2009 | Positive | - | 77 |
| 25_40 | Unknown | India | Unknown | Unknown | - | 77 |
| 25_52 | Cerebrospinal fluid | Chandigarh, India | 1996 | Negative | - | 77 |
| 25_53 | Cerebrospinal fluid | Karnataka, India | 1996 | Negative | - | 77 |
| 25_61 | Cerebrospinal fluid | Calcutta, India | 1996 | Positive | - | 77 |
| 25_62 | Cerebrospinal fluid | Karnataka, India | 1996 | Negative | - | 77 |
| **25_63*** | Cerebrospinal fluid | Karnataka, India | 1996 | Positive | - | 77 |
| 25_84 | Blood | Chandigarh, India | 1998 | Negative | - | 77 |
| 4_9 | Clinical | Khon Kaen, Thailand | Unknown | Unknown | 52 | 82 |
| WH001 | Cerebrospinal fluid | Shanghai, China | 2000 | Negative | - | 93 |
| 25_228 | Cerebrospinal fluid | Chandigarh, India | 2005 | Positive | - | 93 |
| 25_237 | Cerebrospinal fluid | Chandigarh, India | 2005 | Positive | - | 93 |
| 25_239 | Cerebrospinal fluid | Chandigarh, India | 1999 | Positive | - | 93 |
| 25_261 | Cerebrospinal fluid | Chandigarh, India | 2005 | Positive | - | 93 |
| 25_272 | Bronchoalveolar lavage | Chandigarh, India | 2006 | Positive | - | 93 |
| 25_277 | Cerebrospinal fluid | Chandigarh, India | 2006 | Positive | - | 93 |
| 25_290 | Cerebrospinal fluid | Chandigarh, India | 2006 | Positive | - | 93 |
| 25_292 | Cerebrospinal fluid | Chandigarh, India | 2006 | Positive | - | 93 |
| 25_299 | Cerebrospinal fluid | Chandigarh, India | 2007 | Negative | - | 93 |
| 25_302 | Cerebrospinal fluid | Chandigarh, India | 2007 | Positive | - | 93 |
| 25_304 | Cerebrospinal fluid | Chandigarh, India | 2007 | Positive | - | 93 |
| 25_308 | Cerebrospinal fluid | Chandigarh, India | 2007 | Positive | - | 93 |
| 25_312 | Cerebrospinal fluid | Chandigarh, India | 2007 | Positive | - | 93 |
| 25_313 | Cerebrospinal fluid | Chandigarh, India | 2007 | Positive | - | 93 |
| 25_334 | Cerebrospinal fluid | Chandigarh, India | 2008 | Positive | - | 93 |
| 25_336 | Cerebrospinal fluid | Chandigarh, India | 2008 | Positive | - | 93 |
| 25_337 | Cerebrospinal fluid | Chandigarh, India | 2008 | Positive | - | 93 |
| 25_339 | Cerebrospinal fluid | Chandigarh, India | 2008 | Negative | - | 93 |
| 25_341 | Cerebrospinal fluid | Chandigarh, India | 2008 | Positive | - | 93 |
| 25_344 | Bronchoalveolar lavage | Delhi, India | 2008 | Positive | - | 93 |
| 25_356 | Cerebrospinal fluid | Chandigarh, India | 2009 | Positive | - | 93 |
| 25_358 | Cerebrospinal fluid | Chandigarh, India | 2005 | Positive | - | 93 |
| 25_367 | Cerebrospinal fluid | Chandigarh, India | 2009 | Positive | - | 93 |
| 25_368 | Cerebrospinal fluid | Chandigarh, India | 2009 | Positive | - | 93 |
| 25_371 | Cerebrospinal fluid | Assam, India | 2009 | Negative | - | 93 |
| 25_49 | Cerebrospinal fluid | Chandigarh, India | 1995 | Negative | - | 93 |
| 25_50 | Cerebrospinal fluid | Chandigarh, India | 1995 | Negative | - | 93 |
| 25_78 | Unknown | India | 1998 | Unknown | - | 93 |
| 25_86 | Cerebrospinal fluid | Karnataka, India | 1998 | Negative | - | 93 |
| 132 | Cerebrospinal fluid | Jakarta, Indonesia | 2007 | Positive | - | 93 |
| 597 | Cerebrospinal fluid | Jakarta, Indonesia | 2006 | Positive | - | 93 |
| **676*^,#^** | Cerebrospinal fluid | Jakarta, Indonesia | 2006 | Positive | - | 93 |
| **1019*** | Cerebrospinal fluid | Jakarta, Indonesia | 2005 | Positive | - | 93 |
| **1051*** | Cerebrospinal fluid | Jakarta, Indonesia | 2006 | Positive | - | 93 |
| **1116*** | Cerebrospinal fluid | Jakarta, Indonesia | 2006 | Positive | - | 93 |
| **1200*** | Cerebrospinal fluid | Jakarta, Indonesia | Unknown | Positive | - | 93 |
| **1206*^,#^** | Cerebrospinal fluid | Jakarta, Indonesia | 2006 | Positive | - | 93 |
| **1336*** | Cerebrospinal fluid | Jakarta, Indonesia | 2006 | Positive | - | 93 |
| **1462*** | Cerebrospinal fluid | Jakarta, Indonesia | 2006 | Positive | - | 93 |
| **1571*^,#^** | Cerebrospinal fluid | Jakarta, Indonesia | 2006 | Positive | - | 93 |
| 2126 | Cerebrospinal fluid | Jakarta, Indonesia | Unknown | Positive | - | 93 |
| **3281*** | Unknown | Jakarta, Indonesia | Unknown | Unknown | - | 93 |
| **3400*^,#^** | Cerebrospinal fluid | Jakarta, Indonesia | 2005 | Positive | - | 93 |
| 3594 | Cerebrospinal fluid | Jakarta, Indonesia | 2006 | Positive | - | 93 |
| 3634 | Cerebrospinal fluid | Jakarta, Indonesia | 2006 | Positive | - | 93 |
| **1048*^,#^** | Unknown | Jakarta, Indonesia | Unknown | Unknown | - | 93 |
| **Jakarta 1051*** | Cerebrospinal fluid | Jakarta, Indonesia | 2006 | Positive | - | 93 |
| 481_03 | Cerebrospinal fluid | Kuwait city, Kuwait | 2003 | Positive | - | 93 |
| CN5007 | Cerebrospinal fluid | Chiang Rai, Thailand | 2007 | Positive | 47 | 93 |
| 1291-09 | Blood | Patnai, Thailand | Unknown | Negative | 47 | 93 |
| **CM35*** | Cerebrospinal fluid | Ubon Ratchatani, Thailand | 2002 | Positive | 47 | 93 |
| **25_17^#^** | Blood | Chandigarh, India | 1989 | Negative | - | 174 |
| 25_240 | Cerebrospinal fluid | Chandigarh, India | 2005 | Negative | - | 174 |
| 25_296 | Cerebrospinal fluid | Chandigarh, India | 2007 | Positive | - | 174 |
| 2365_08 ^IN^ | Cerebrospinal fluid | Kuwait city, Kuwait | 2008 | Negative | - | 174 |
| 200_16 | Lymph node biopsy | Kuwait city, Kuwait | 1996 | Positive | - | 175 |
| 25_33 | Cerebrospinal fluid | Chandigarh, India | 1991 | Negative | - | 177 |
| Cr2231 | Cerebrospinal fluid | Jakarta, Indonesia | 2004 | Positive | - | 177 |
| Cr755 | Cerebrospinal fluid | Jakarta, Indonesia | 2004 | Positive | - | 177 |
| CrOE | Cerebrospinal fluid | Jakarta, Indonesia | Unknown | Negative | - | 177 |
| 177/02 | Wound swab | Kuwait city, Kuwait | 2002 | Negative | - | 185 |
| WH095 | Urine | Shanghai, China | 2001 | Negative | - | 186 |
| 25_340 | Cerebrospinal fluid | Chandigarh, India | 2008 | Negative | - | 187 |
| 25_311 | Cerebrospinal fluid | Chandigarh, India | 2007 | Negative | - | 189 |
| WH069 | Cerebrospinal fluid | Shanghai, China | 2001 | Negative | - | 191 |
| 1589_04 | Cerebrospinal fluid | Kuwait city, Kuwait | 2004 | Negative | - | 192 |
| WH071 | Cerebrospinal fluid | Shanghai, China | 1998 | Negative | - | 194 |
| WH123 | Cerebrospinal fluid | Guangdong, China | 2007 | Positive | - | 194 |
| WH132 | Cerebrospinal fluid | Guangdong, China | 2007 | Positive | - | 195 |

^ID^ : isolate from patient originating from India

^IN^ : isolate from patient originating from Indonesia

^MM^ : isolate from patient originating from Myanmar

^PH^ : isolate from patient originating from Philippines

^SD^ : isolate from patient originating from Sudan

* : isolate with MIC ≥ 16 of 5FC

^#^ : isolate with MIC ≥ 16 of FLU

^¶^ : isolate with MIC ≥ 0.5 of VOR

Table B. Environmental *Cryptococcus* *neoformans* var. *grubii* αA isolates from Thailand and Japan.

| Strain | Sample | Location | Year | STs (in this study) |
| --- | --- | --- | --- | --- |
| 109A | Avian guano | Chiang Mai, Thailand | Unknown | 4 |
| 109C | Avian guano | Chiang Mai, Thailand | Unknown | 4 |
| 110C | Avian guano | Chiang Mai, Thailand | Unknown | 4 |
| 110D | Avian guano | Chiang Mai, Thailand | Unknown | 4 |
| 130C | Avian guano | Chiang Mai, Thailand | Unknown | 4 |
| 96B | Avian guano | Chiang Mai, Thailand | Unknown | 4 |
| D12 | Avian guano | Chiang Mai, Thailand | 2000 | 4 |
| D14 | Avian guano | Chiang Mai, Thailand | 2000 | 4 |
| D2 | Avian guano | Chiang Mai, Thailand | 2000 | 4 |
| D27 | Avian guano | Chiang Mai, Thailand | 2000 | 4 |
| D28 | Avian guano | Chiang Mai, Thailand | 2000 | 4 |
| D3 | Avian guano | Chiang Mai, Thailand | 2000 | 4 |
| D31 | Avian guano | Chiang Mai, Thailand | 2000 | 4 |
| D43 | Avian guano | Chiang Mai, Thailand | 2000 | 4 |
| D46 | Avian guano | Chiang Mai, Thailand | 2000 | 4 |
| D69 | Avian guano | Chiang Mai, Thailand | 2000 | 4 |
| D71 | Avian guano | Chiang Mai, Thailand | 2000 | 4 |
| D73 | Avian guano | Chiang Mai, Thailand | 2000 | 4 |
| PG21 | Avian guano | Chiang Mai, Thailand | 2000 | 4 |
| PG26 | Avian guano | Chiang Mai, Thailand | 2000 | 4 |
| PG3 | Avian guano | Chiang Mai, Thailand | 2000 | 4 |
| PG46 | Avian guano | Chiang Mai, Thailand | 2000 | 4 |
| 40 | Avian guano | Chiang Mai, Thailand | Unknown | 5 |
| 189E | Avian guano | Chiang Mai, Thailand | Unknown | 5 |
| D16 | Avian guano | Chiang Mai, Thailand | 2000 | 5 |
| D4 | Avian guano | Chiang Mai, Thailand | 2000 | 5 |
| D6 | Avian guano | Chiang Mai, Thailand | 2000 | 5 |
| PG37 | Avian guano | Chiang Mai, Thailand | 2000 | 5 |
| 44 | Avian guano | Chiang Mai, Thailand | Unknown | 6 |
| 2551-07 | Avian guano | Chiang Mai, Thailand | Unknown | 6 |
| D1 | Avian guano | Chiang Mai, Thailand | 2000 | 6 |
| D18 | Avian guano | Chiang Mai, Thailand | 2000 | 6 |
| D21 | Avian guano | Chiang Mai, Thailand | 2000 | 6 |
| D22 | Avian guano | Chiang Mai, Thailand | 2000 | 6 |
| D26 | Avian guano | Chiang Mai, Thailand | 2000 | 6 |
| D30 | Avian guano | Chiang Mai, Thailand | 2000 | 6 |
| D33 | Avian guano | Chiang Mai, Thailand | 2000 | 6 |
| D34 | Avian guano | Chiang Mai, Thailand | 2000 | 6 |
| D35 | Avian guano | Chiang Mai, Thailand | 2000 | 6 |
| D36 | Avian guano | Chiang Mai, Thailand | 2000 | 6 |
| D41 | Avian guano | Chiang Mai, Thailand | 2000 | 6 |
| D64 | Avian guano | Chiang Mai, Thailand | 2000 | 6 |
| D76 | Avian guano | Chiang Mai, Thailand | 2000 | 6 |
| PG1 | Avian guano | Chiang Mai, Thailand | 2000 | 6 |
| PG2 | Avian guano | Chiang Mai, Thailand | 2000 | 6 |
| PG32 | Avian guano | Chiang Mai, Thailand | 2000 | 6 |
| D15 | Avian guano | Chiang Mai, Thailand | 2000 | 31 |
| D17 | Avian guano | Chiang Mai, Thailand | 2000 | 31 |
| D19 | Avian guano | Chiang Mai, Thailand | 2000 | 31 |
| 34 | Avian guano | Chiang Mai, Thailand | Unknown | 53 |
| D9 | Avian guano | Chiang Mai, Thailand | 2000 | 141 |
| D45 | Avian guano | Chiang Mai, Thailand | 2000 | 175 |
| D42 | Avian guano | Chiang Mai, Thailand | 2000 | 176 |
| 110A | Avian guano | Chiang Mai, Thailand | Unknown | 185 |
| 110E | Avian guano | Chiang Mai, Thailand | Unknown | 188 |
| 130D | Avian guano | Chiang Mai, Thailand | Unknown | 190 |
| D44 | Avian guano | Chiang Mai, Thailand | 2000 | 193 |
| 9211 | Avian guano | Tokyo, Japan | 2005 | 5 |
| 9212 | Avian guano | Tokyo, Japan | 2005 | 5 |
| 9254 | Avian guano | Tokyo, Japan | 2005 | 5 |
| 9255 | Avian guano | Tokyo, Japan | 2005 | 5 |
| 9258 | Avian guano | Tokyo, Japan | 2005 | 5 |
| 9259 | Avian guano | Tokyo, Japan | 2005 | 5 |
| 9260 | Avian guano | Tokyo, Japan | 2005 | 5 |
| 9261 | Avian guano | Tokyo, Japan | 2005 | 5 |
| 9257 | Avian guano | Tokyo, Japan | 2005 | 23 |
| 9256 | Avian guano | Tokyo, Japan | 2005 | 31 |

Table C. Accession number of sequences from each locus of Asian *C*. *neoformans* var. *grubii* isolates.

| Isolate | *CAP59* | *GPD1* | IGS1 | *LAC1* | *PLB1* | *SOD1* | URA5 |
| --- | --- | --- | --- | --- | --- | --- | --- |
| WH001 | KC529683 | KC530158 | KC530634 | KC531109 | KC531584 | KC532059 | KC532534 |
| WH003 | KC529684 | KC530159 | KC530635 | KC531110 | KC531585 | KC532060 | KC532535 |
| WH004 | KC529685 | KC530160 | KC530636 | KC531111 | KC531586 | KC532061 | KC532536 |
| WH005 | KC529686 | KC530161 | KC530637 | KC531112 | KC531587 | KC532062 | KC532537 |
| WH006 | KC529687 | KC530162 | KC530638 | KC531113 | KC531588 | KC532063 | KC532538 |
| WH007 | KC529688 | KC530163 | KC530639 | KC531114 | KC531589 | KC532064 | KC532539 |
| WH008 | KC529689 | KC530164 | KC530640 | KC531115 | KC531590 | KC532065 | KC532540 |
| WH011 | KC529690 | KC530165 | KC530641 | KC531116 | KC531591 | KC532066 | KC532541 |
| WH012 | KC529691 | KC530166 | KC530642 | KC531117 | KC531592 | KC532067 | KC532542 |
| WH013 | KC529692 | KC530167 | KC530643 | KC531118 | KC531593 | KC532068 | KC532543 |
| WH014 | KC529693 | KC530168 | KC530644 | KC531119 | KC531594 | KC532069 | KC532544 |
| WH015 | KC529694 | KC530169 | KC530645 | KC531120 | KC531595 | KC532070 | KC532545 |
| WH016 | KC529695 | KC530170 | KC530646 | KC531121 | KC531596 | KC532071 | KC532546 |
| WH017 | KC529696 | KC530171 | KC530647 | KC531122 | KC531597 | KC532072 | KC532547 |
| WH018 | KC529697 | KC530172 | KC530648 | KC531123 | KC531598 | KC532073 | KC532548 |
| WH019 | KC529698 | KC530173 | KC530649 | KC531124 | KC531599 | KC532074 | KC532549 |
| WH020 | KC529699 | KC530174 | KC530650 | KC531125 | KC531600 | KC532075 | KC532550 |
| WH022 | KC529700 | KC530175 | KC530651 | KC531126 | KC531601 | KC532076 | KC532551 |
| WH023 | KC529701 | KC530176 | KC530652 | KC531127 | KC531602 | KC532077 | KC532552 |
| WH025 | KC529702 | KC530177 | KC530653 | KC531128 | KC531603 | KC532078 | KC532553 |
| WH026 | KC529703 | KC530178 | KC530654 | KC531129 | KC531604 | KC532079 | KC532554 |
| WH027 | KC529704 | KC530179 | KC530655 | KC531130 | KC531605 | KC532080 | KC532555 |
| WH028 | KC529705 | KC530180 | KC530656 | KC531131 | KC531606 | KC532081 | KC532556 |
| WH030 | KC529706 | KC530181 | KC530657 | KC531132 | KC531607 | KC532082 | KC532557 |
| WH031 | KC529707 | KC530182 | KC530658 | KC531133 | KC531608 | KC532083 | KC532558 |
| WH033 | KC529708 | KC530183 | KC530659 | KC531134 | KC531609 | KC532084 | KC532559 |
| WH034 | KC529709 | KC530184 | KC530660 | KC531135 | KC531610 | KC532085 | KC532560 |
| WH035 | KC529710 | KC530185 | KC530661 | KC531136 | KC531611 | KC532086 | KC532561 |
| WH036 | KC529711 | KC530186 | KC530662 | KC531137 | KC531612 | KC532087 | KC532562 |
| WH037 | KC529712 | KC530187 | KC530663 | KC531138 | KC531613 | KC532088 | KC532563 |
| WH040 | KC529713 | KC530188 | KC530664 | KC531139 | KC531614 | KC532089 | KC532564 |
| WH041 | KC529714 | KC530189 | KC530665 | KC531140 | KC531615 | KC532090 | KC532565 |
| WH042 | KC529715 | KC530190 | KC530666 | KC531141 | KC531616 | KC532091 | KC532566 |
| WH044 | KC529716 | KC530191 | KC530667 | KC531142 | KC531617 | KC532092 | KC532567 |
| WH047 | KC529717 | KC530192 | KC530668 | KC531143 | KC531618 | KC532093 | KC532568 |
| WH050 | KC529718 | KC530193 | KC530669 | KC531144 | KC531619 | KC532094 | KC532569 |
| WH051 | KC529719 | KC530194 | KC530670 | KC531145 | KC531620 | KC532095 | KC532570 |
| WH054 | KC529720 | KC530195 | KC530671 | KC531146 | KC531621 | KC532096 | KC532571 |
| WH055 | KC529721 | KC530196 | KC530672 | KC531147 | KC531622 | KC532097 | KC532572 |
| WH057 | KC529722 | KC530197 | KC530673 | KC531148 | KC531623 | KC532098 | KC532573 |
| WH058 | KC529723 | KC530198 | KC530674 | KC531149 | KC531624 | KC532099 | KC532574 |
| WH061 | KC529724 | KC530199 | KC530675 | KC531150 | KC531625 | KC532100 | KC532575 |
| WH062 | KC529725 | KC530200 | KC530676 | KC531151 | KC531626 | KC532101 | KC532576 |
| WH063 | KC529726 | KC530201 | KC530677 | KC531152 | KC531627 | KC532102 | KC532577 |
| WH066 | KC529727 | KC530202 | KC530678 | KC531153 | KC531628 | KC532103 | KC532578 |
| WH067 | KC529728 | KC530203 | KC530679 | KC531154 | KC531629 | KC532104 | KC532579 |
| WH068 | KC529729 | KC530204 | KC530680 | KC531155 | KC531630 | KC532105 | KC532580 |
| WH069 | KC529730 | KC530205 | KC530681 | KC531156 | KC531631 | KC532106 | KC532581 |
| WH070 | KC529731 | KC530206 | KC530682 | KC531157 | KC531632 | KC532107 | KC532582 |
| WH071 | KC529732 | KC530207 | KC530683 | KC531158 | KC531633 | KC532108 | KC532583 |
| WH072 | KC529733 | KC530208 | KC530684 | KC531159 | KC531634 | KC532109 | KC532584 |
| WH073 | KC529734 | KC530209 | KC530685 | KC531160 | KC531635 | KC532110 | KC532585 |
| WH075 | KC529735 | KC530210 | KC530686 | KC531161 | KC531636 | KC532111 | KC532586 |
| WH076 | KC529736 | KC530211 | KC530687 | KC531162 | KC531637 | KC532112 | KC532587 |
| WH077 | KC529737 | KC530212 | KC530688 | KC531163 | KC531638 | KC532113 | KC532588 |
| WH078 | KC529738 | KC530213 | KC530689 | KC531164 | KC531639 | KC532114 | KC532589 |
| WH079 | KC529739 | KC530214 | KC530690 | KC531165 | KC531640 | KC532115 | KC532590 |
| WH080 | KC529740 | KC530215 | KC530691 | KC531166 | KC531641 | KC532116 | KC532591 |
| WH082 | KC529741 | KC530216 | KC530692 | KC531167 | KC531642 | KC532117 | KC532592 |
| WH083 | KC529742 | KC530217 | KC530693 | KC531168 | KC531643 | KC532118 | KC532593 |
| WH091 | KC529743 | KC530218 | KC530694 | KC531169 | KC531644 | KC532119 | KC532594 |
| WH093 | KC529744 | KC530219 | KC530695 | KC531170 | KC531645 | KC532120 | KC532595 |
| WH094 | KC529745 | KC530220 | KC530696 | KC531171 | KC531646 | KC532121 | KC532596 |
| WH095 | KC529746 | KC530221 | KC530697 | KC531172 | KC531647 | KC532122 | KC532597 |
| WH096 | KC529747 | KC530222 | KC530698 | KC531173 | KC531648 | KC532123 | KC532598 |
| WH098 | KC529748 | KC530223 | KC530699 | KC531174 | KC531649 | KC532124 | KC532599 |
| WH101 | KC529749 | KC530224 | KC530700 | KC531175 | KC531650 | KC532125 | KC532600 |
| WH102 | KC529750 | KC530225 | KC530701 | KC531176 | KC531651 | KC532126 | KC532601 |
| WH104 | KC529751 | KC530226 | KC530702 | KC531177 | KC531652 | KC532127 | KC532602 |
| WH105 | KC529752 | KC530227 | KC530703 | KC531178 | KC531653 | KC532128 | KC532603 |
| WH106 | KC529753 | KC530228 | KC530704 | KC531179 | KC531654 | KC532129 | KC532604 |
| WH108 | KC529754 | KC530229 | KC530705 | KC531180 | KC531655 | KC532130 | KC532605 |
| WH113 | KC529755 | KC530230 | KC530706 | KC531181 | KC531656 | KC532131 | KC532606 |
| WH114 | KC529756 | KC530231 | KC530707 | KC531182 | KC531657 | KC532132 | KC532607 |
| WH115 | KC529757 | KC530232 | KC530708 | KC531183 | KC531658 | KC532133 | KC532608 |
| WH117 | KC529758 | KC530233 | KC530709 | KC531184 | KC531659 | KC532134 | KC532609 |
| WH118 | KC529759 | KC530234 | KC530710 | KC531185 | KC531660 | KC532135 | KC532610 |
| WH119 | KC529760 | KC530235 | KC530711 | KC531186 | KC531661 | KC532136 | KC532611 |
| WH120 | KC529761 | KC530236 | KC530712 | KC531187 | KC531662 | KC532137 | KC532612 |
| WH121 | KC529762 | KC530237 | KC530713 | KC531188 | KC531663 | KC532138 | KC532613 |
| WH122 | KC529763 | KC530238 | KC530714 | KC531189 | KC531664 | KC532139 | KC532614 |
| WH123 | KC529764 | KC530239 | KC530715 | KC531190 | KC531665 | KC532140 | KC532615 |
| WH124 | KC529765 | KC530240 | KC530716 | KC531191 | KC531666 | KC532141 | KC532616 |
| WH125 | KC529766 | KC530241 | KC530717 | KC531192 | KC531667 | KC532142 | KC532617 |
| WH126 | KC529767 | KC530242 | KC530718 | KC531193 | KC531668 | KC532143 | KC532618 |
| WH132 | KC529768 | KC530243 | KC530719 | KC531194 | KC531669 | KC532144 | KC532619 |
| HK_04 | KC529769 | KC530244 | KC530720 | KC531195 | KC531670 | KC532145 | KC532620 |
| HK_05 | KC529770 | KC530245 | KC530721 | KC531196 | KC531671 | KC532146 | KC532621 |
| HK_06 | KC529771 | KC530246 | KC530722 | KC531197 | KC531672 | KC532147 | KC532622 |
| HK_01 | KC529772 | KC530247 | KC530723 | KC531198 | KC531673 | KC532148 | KC532623 |
| HK_07 | KC529773 | KC530248 | KC530724 | KC531199 | KC531674 | KC532149 | KC532624 |
| HK_08 | KC529774 | KC530249 | KC530725 | KC531200 | KC531675 | KC532150 | KC532625 |
| HK_09 | KC529775 | KC530250 | KC530726 | KC531201 | KC531676 | KC532151 | KC532626 |
| HK_10 | KC529776 | KC530251 | KC530727 | KC531202 | KC531677 | KC532152 | KC532627 |
| HK_11 | KC529777 | KC530252 | KC530728 | KC531203 | KC531678 | KC532153 | KC532628 |
| HK_12 | KC529778 | KC530253 | KC530729 | KC531204 | KC531679 | KC532154 | KC532629 |
| HK_13 | KC529779 | KC530254 | KC530730 | KC531205 | KC531680 | KC532155 | KC532630 |
| HK-14 | KC529780 | KC530255 | KC530731 | KC531206 | KC531681 | KC532156 | KC532631 |
| HK-03 | KC529781 | KC530256 | KC530732 | KC531207 | KC531682 | KC532157 | KC532632 |
| HK_02 | KC529782 | KC530257 | KC530733 | KC531208 | KC531683 | KC532158 | KC532633 |
| 25_104 | KC529783 | KC530258 | KC530734 | KC531209 | KC531684 | KC532159 | KC532634 |
| 25_105 | KC529784 | KC530259 | KC530735 | KC531210 | KC531685 | KC532160 | KC532635 |
| 25_110 | KC529785 | KC530260 | KC530736 | KC531211 | KC531686 | KC532161 | KC532636 |
| 25_14 | KC529786 | KC530261 | KC530737 | KC531212 | KC531687 | KC532162 | KC532637 |
| 25_17 | KC529787 | KC530262 | KC530738 | KC531213 | KC531688 | KC532163 | KC532638 |
| 25_18 | KC529788 | KC530263 | KC530739 | KC531214 | KC531689 | KC532164 | KC532639 |
| 25_228 | KC529789 | KC530264 | KC530740 | KC531215 | KC531690 | KC532165 | KC532640 |
| 25_229 | KC529790 | KC530265 | KC530741 | KC531216 | KC531691 | KC532166 | KC532641 |
| 25_237 | KC529791 | KC530266 | KC530742 | KC531217 | KC531692 | KC532167 | KC532642 |
| 25_239 | KC529792 | KC530267 | KC530743 | KC531218 | KC531693 | KC532168 | KC532643 |
| 25_240 | KC529793 | KC530268 | KC530744 | KC531219 | KC531694 | KC532169 | KC532644 |
| 25_244 | KC529794 | KC530269 | KC530745 | KC531220 | KC531695 | KC532170 | KC532645 |
| 25_261 | KC529795 | KC530270 | KC530746 | KC531221 | KC531696 | KC532171 | KC532646 |
| 25_266 | KC529796 | KC530271 | KC530747 | KC531222 | KC531697 | KC532172 | KC532647 |
| 25_272 | KC529797 | KC530272 | KC530748 | KC531223 | KC531698 | KC532173 | KC532648 |
| 25_277 | KC529798 | KC530273 | KC530749 | KC531224 | KC531699 | KC532174 | KC532649 |
| 25_290 | KC529799 | KC530274 | KC530750 | KC531225 | KC531700 | KC532175 | KC532650 |
| 25_291 | KC529800 | KC530275 | KC530751 | KC531226 | KC531701 | KC532176 | KC532651 |
| 25_292 | KC529801 | KC530276 | KC530752 | KC531227 | KC531702 | KC532177 | KC532652 |
| 25_296 | KC529802 | KC530277 | KC530753 | KC531228 | KC531703 | KC532178 | KC532653 |
| 25_298 | KC529803 | KC530278 | KC530754 | KC531229 | KC531704 | KC532179 | KC532654 |
| 25_299 | KC529804 | KC530279 | KC530755 | KC531230 | KC531705 | KC532180 | KC532655 |
| 25_302 | KC529805 | KC530280 | KC530756 | KC531231 | KC531706 | KC532181 | KC532656 |
| 25_304 | KC529806 | KC530281 | KC530757 | KC531232 | KC531707 | KC532182 | KC532657 |
| 25_308 | KC529807 | KC530282 | KC530758 | KC531233 | KC531708 | KC532183 | KC532658 |
| 25_311 | KC529808 | KC530283 | KC530759 | KC531234 | KC531709 | KC532184 | KC532659 |
| 25_312 | KC529809 | KC530284 | KC530760 | KC531235 | KC531710 | KC532185 | KC532660 |
| 25_313 | KC529810 | KC530285 | KC530761 | KC531236 | KC531711 | KC532186 | KC532661 |
| 25_316 | KC529811 | KC530286 | KC530762 | KC531237 | KC531712 | KC532187 | KC532662 |
| 25_328 | KC529812 | KC530287 | KC530763 | KC531238 | KC531713 | KC532188 | KC532663 |
| 25_33 | KC529813 | KC530288 | KC530764 | KC531239 | KC531714 | KC532189 | KC532664 |
| 25_334 | KC529814 | KC530289 | KC530765 | KC531240 | KC531715 | KC532190 | KC532665 |
| 25_336 | KC529815 | KC530290 | KC530766 | KC531241 | KC531716 | KC532191 | KC532666 |
| 25_337 | KC529816 | KC530291 | KC530767 | KC531242 | KC531717 | KC532192 | KC532667 |
| 25_339 | KC529817 | KC530292 | KC530768 | KC531243 | KC531718 | KC532193 | KC532668 |
| 25_340 | KC529818 | KC530293 | KC530769 | KC531244 | KC531719 | KC532194 | KC532669 |
| 25_341 | KC529819 | KC530294 | KC530770 | KC531245 | KC531720 | KC532195 | KC532670 |
| 25_344 | KC529820 | KC530295 | KC530771 | KC531246 | KC531721 | KC532196 | KC532671 |
| 25_355 | KC529821 | KC530296 | KC530772 | KC531247 | KC531722 | KC532197 | KC532672 |
| 25_356 | KC529822 | KC530297 | KC530773 | KC531248 | KC531723 | KC532198 | KC532673 |
| 25_357 | KC529823 | KC530298 | KC530774 | KC531249 | KC531724 | KC532199 | KC532674 |
| 25_358 | KC529824 | KC530299 | KC530775 | KC531250 | KC531725 | KC532200 | KC532675 |
| 25_365 | KC529825 | KC530300 | KC530776 | KC531251 | KC531726 | KC532201 | KC532676 |
| 25_367 | KC529826 | KC530301 | KC530777 | KC531252 | KC531727 | KC532202 | KC532677 |
| 25_368 | KC529827 | KC530302 | KC530778 | KC531253 | KC531728 | KC532203 | KC532678 |
| 25_369 | KC529828 | KC530303 | KC530779 | KC531254 | KC531729 | KC532204 | KC532679 |
| 25_370 | KC529829 | KC530304 | KC530780 | KC531255 | KC531730 | KC532205 | KC532680 |
| 25_371 | KC529830 | KC530305 | KC530781 | KC531256 | KC531731 | KC532206 | KC532681 |
| 25_372 | KC529831 | KC530306 | KC530782 | KC531257 | KC531732 | KC532207 | KC532682 |
| 25_373 | KC529832 | KC530307 | KC530783 | KC531258 | KC531733 | KC532208 | KC532683 |
| 25_40 | KC529833 | KC530308 | KC530784 | KC531259 | KC531734 | KC532209 | KC532684 |
| 25_49 | KC529834 | KC530309 | KC530785 | KC531260 | KC531735 | KC532210 | KC532685 |
| 25_50 | KC529835 | KC530310 | KC530786 | KC531261 | KC531736 | KC532211 | KC532686 |
| 25_52 | KC529836 | KC530311 | KC530787 | KC531262 | KC531737 | KC532212 | KC532687 |
| 25_53 | KC529837 | KC530312 | KC530788 | KC531263 | KC531738 | KC532213 | KC532688 |
| 25_61 | KC529838 | KC530313 | KC530789 | KC531264 | KC531739 | KC532214 | KC532689 |
| 25_62 | KC529839 | KC530314 | KC530790 | KC531265 | KC531740 | KC532215 | KC532690 |
| 25_63 | KC529840 | KC530315 | KC530791 | KC531266 | KC531741 | KC532216 | KC532691 |
| 25_78 | KC529841 | KC530316 | KC530792 | KC531267 | KC531742 | KC532217 | KC532692 |
| 25_84 | KC529842 | KC530317 | KC530793 | KC531268 | KC531743 | KC532218 | KC532693 |
| 25_86 | KC529843 | KC530318 | KC530794 | KC531269 | KC531744 | KC532219 | KC532694 |
| 132 | KC529844 | KC530319 | KC530795 | KC531270 | KC531745 | KC532220 | KC532695 |
| 1336 | KC529845 | KC530320 | KC530796 | KC531271 | KC531746 | KC532221 | KC532696 |
| 1462 | KC529846 | KC530321 | KC530797 | KC531272 | KC531747 | KC532222 | KC532697 |
| 1571 | KC529847 | KC530322 | KC530798 | KC531273 | KC531748 | KC532223 | KC532698 |
| 1019 | KC529848 | KC530323 | KC530799 | KC531274 | KC531749 | KC532224 | KC532699 |
| 1048 | KC529849 | KC530324 | KC530800 | KC531275 | KC531750 | KC532225 | KC532700 |
| 1051 | KC529850 | KC530325 | KC530801 | KC531276 | KC531751 | KC532226 | KC532701 |
| Jakarta_1051 | KC529851 | KC530326 | KC530802 | KC531277 | KC531752 | KC532227 | KC532702 |
| 1116 | KC529852 | KC530327 | KC530803 | KC531278 | KC531753 | KC532228 | KC532703 |
| 1200 | KC529853 | KC530328 | KC530804 | KC531279 | KC531754 | KC532229 | KC532704 |
| 1206 | KC529854 | KC530329 | KC530805 | KC531280 | KC531755 | KC532230 | KC532705 |
| 2126 | KC529855 | KC530330 | KC530806 | KC531281 | KC531756 | KC532231 | KC532706 |
| 2339 | KC529856 | KC530331 | KC530807 | KC531282 | KC531757 | KC532232 | KC532707 |
| 2478 | KC529857 | KC530332 | KC530808 | KC531283 | KC531758 | KC532233 | KC532708 |
| 2594 | KC529858 | KC530333 | KC530809 | KC531284 | KC531759 | KC532234 | KC532709 |
| 2597 | KC529859 | KC530334 | KC530810 | KC531285 | KC531760 | KC532235 | KC532710 |
| 2606 | KC529860 | KC530335 | KC530811 | KC531286 | KC531761 | KC532236 | KC532711 |
| 264 | KC529861 | KC530336 | KC530812 | KC531287 | KC531762 | KC532237 | KC532712 |
| 267 | KC529862 | KC530337 | KC530813 | KC531288 | KC531763 | KC532238 | KC532713 |
| 268 | KC529863 | KC530338 | KC530814 | KC531289 | KC531764 | KC532239 | KC532714 |
| 3187 | KC529864 | KC530339 | KC530815 | KC531290 | KC531765 | KC532240 | KC532715 |
| 328 | KC529865 | KC530340 | KC530816 | KC531291 | KC531766 | KC532241 | KC532716 |
| 3281 | KC529866 | KC530341 | KC530817 | KC531292 | KC531767 | KC532242 | KC532717 |
| 3400 | KC529867 | KC530342 | KC530818 | KC531293 | KC531768 | KC532243 | KC532718 |
| 3594 | KC529868 | KC530343 | KC530819 | KC531294 | KC531769 | KC532244 | KC532719 |
| 3634 | KC529869 | KC530344 | KC530820 | KC531295 | KC531770 | KC532245 | KC532720 |
| 544 | KC529870 | KC530345 | KC530821 | KC531296 | KC531771 | KC532246 | KC532721 |
| 597 | KC529871 | KC530346 | KC530822 | KC531297 | KC531772 | KC532247 | KC532722 |
| 612 | KC529872 | KC530347 | KC530823 | KC531298 | KC531773 | KC532248 | KC532723 |
| 676 | KC529873 | KC530348 | KC530824 | KC531299 | KC531774 | KC532249 | KC532724 |
| 778 | KC529874 | KC530349 | KC530825 | KC531300 | KC531775 | KC532250 | KC532725 |
| Jakarta_P | KC529875 | KC530350 | KC530826 | KC531301 | KC531776 | KC532251 | KC532726 |
| Jakarta_H | KC529876 | KC530351 | KC530827 | KC531302 | KC531777 | KC532252 | KC532727 |
| Jakarta_KT | KC529877 | KC530352 | KC530828 | KC531303 | KC531778 | KC532253 | KC532728 |
| Jakarta_KLT | KC529878 | KC530353 | KC530829 | KC531304 | KC531779 | KC532254 | KC532729 |
| Jakarta_RLT | KC529879 | KC530354 | KC530830 | KC531305 | KC531780 | KC532255 | KC532730 |
| Jakarta | KC529880 | KC530355 | KC530831 | KC531306 | KC531781 | KC532256 | KC532731 |
| CR0E | KC529881 | KC530356 | KC530832 | KC531307 | KC531782 | KC532257 | KC532732 |
| CR2231 | KC529882 | KC530357 | KC530833 | KC531308 | KC531783 | KC532258 | KC532733 |
| CR755 | KC529883 | KC530358 | KC530834 | KC531309 | KC531784 | KC532259 | KC532734 |
| 9104 | KC529884 | KC530359 | KC530835 | KC531310 | KC531785 | KC532260 | KC532735 |
| 9106 | KC529885 | KC530360 | KC530836 | KC531311 | KC531786 | KC532261 | KC532736 |
| 9107 | KC529886 | KC530361 | KC530837 | KC531312 | KC531787 | KC532262 | KC532737 |
| 9108 | KC529887 | KC530362 | KC530838 | KC531313 | KC531788 | KC532263 | KC532738 |
| 9111 | KC529888 | KC530363 | KC530839 | KC531314 | KC531789 | KC532264 | KC532739 |
| 9114 | KC529889 | KC530364 | KC530840 | KC531315 | KC531790 | KC532265 | KC532740 |
| 9165 | KC529890 | KC530365 | KC530841 | KC531316 | KC531791 | KC532266 | KC532741 |
| 9166 | KC529891 | KC530366 | KC530842 | KC531317 | KC531792 | KC532267 | KC532742 |
| 9167 | KC529892 | KC530367 | KC530843 | KC531318 | KC531793 | KC532268 | KC532743 |
| 9170 | KC529893 | KC530368 | KC530844 | KC531319 | KC531794 | KC532269 | KC532744 |
| 9172 | KC529894 | KC530369 | KC530845 | KC531320 | KC531795 | KC532270 | KC532745 |
| 9173 | KC529895 | KC530370 | KC530846 | KC531321 | KC531796 | KC532271 | KC532746 |
| 9174 | KC529896 | KC530371 | KC530847 | KC531322 | KC531797 | KC532272 | KC532747 |
| 9179 | KC529897 | KC530372 | KC530848 | KC531323 | KC531798 | KC532273 | KC532748 |
| 9197 | KC529898 | KC530373 | KC530849 | KC531324 | KC531799 | KC532274 | KC532749 |
| 9198 | KC529899 | KC530374 | KC530850 | KC531325 | KC531800 | KC532275 | KC532750 |
| 9199 | KC529900 | KC530375 | KC530851 | KC531326 | KC531801 | KC532276 | KC532751 |
| 9204 | KC529901 | KC530376 | KC530852 | KC531327 | KC531802 | KC532277 | KC532752 |
| 9205 | KC529902 | KC530377 | KC530853 | KC531328 | KC531803 | KC532278 | KC532753 |
| 9211 | KC529903 | KC530378 | KC530854 | KC531329 | KC531804 | KC532279 | KC532754 |
| 9212 | KC529904 | KC530379 | KC530855 | KC531330 | KC531805 | KC532280 | KC532755 |
| 9213 | KC529905 | KC530380 | KC530856 | KC531331 | KC531806 | KC532281 | KC532756 |
| 9217 | KC529906 | KC530381 | KC530857 | KC531332 | KC531807 | KC532282 | KC532757 |
| 9237 | KC529907 | KC530382 | KC530858 | KC531333 | KC531808 | KC532283 | KC532758 |
| 9238 | KC529908 | KC530383 | KC530859 | KC531334 | KC531809 | KC532284 | KC532759 |
| 9239 | KC529909 | KC530384 | KC530860 | KC531335 | KC531810 | KC532285 | KC532760 |
| 9251 | KC529910 | KC530385 | KC530861 | KC531336 | KC531811 | KC532286 | KC532761 |
| 9254 | KC529911 | KC530386 | KC530862 | KC531337 | KC531812 | KC532287 | KC532762 |
| 9255 | KC529912 | KC530387 | KC530863 | KC531338 | KC531813 | KC532288 | KC532763 |
| 9256 | KC529913 | KC530388 | KC530864 | KC531339 | KC531814 | KC532289 | KC532764 |
| 9257 | KC529914 | KC530389 | KC530865 | KC531340 | KC531815 | KC532290 | KC532765 |
| 9258 | KC529915 | KC530390 | KC530866 | KC531341 | KC531816 | KC532291 | KC532766 |
| 9259 | KC529916 | KC530391 | KC530867 | KC531342 | KC531817 | KC532292 | KC532767 |
| 9260 | KC529917 | KC530392 | KC530868 | KC531343 | KC531818 | KC532293 | KC532768 |
| 9261 | KC529918 | KC530393 | KC530869 | KC531344 | KC531819 | KC532294 | KC532769 |
| 9263 | KC529919 | KC530394 | KC530870 | KC531345 | KC531820 | KC532295 | KC532770 |
| 9264 | KC529920 | KC530395 | KC530871 | KC531346 | KC531821 | KC532296 | KC532771 |
| 9265 | KC529921 | KC530396 | KC530872 | KC531347 | KC531822 | KC532297 | KC532772 |
| 110_99 | KC529922 | KC530397 | KC530873 | KC531348 | KC531823 | KC532298 | KC532773 |
| 1185_04 | KC529923 | KC530398 | KC530874 | KC531349 | KC531824 | KC532299 | KC532774 |
| 1589_04 | KC529924 | KC530399 | KC530875 | KC531350 | KC531825 | KC532300 | KC532775 |
| 177_02 | KC529925 | KC530400 | KC530876 | KC531351 | KC531826 | KC532301 | KC532776 |
| 200_16 | KC529926 | KC530401 | KC530877 | KC531352 | KC531827 | KC532302 | KC532777 |
| 201_95 | KC529927 | KC530402 | KC530878 | KC531353 | KC531828 | KC532303 | KC532778 |
| 194_96 | KC529928 | KC530403 | KC530879 | KC531354 | KC531829 | KC532304 | KC532779 |
| 2365_08 | KC529929 | KC530404 | KC530880 | KC531355 | KC531830 | KC532305 | KC532780 |
| 481_03 | KC529930 | KC530405 | KC530881 | KC531356 | KC531831 | KC532306 | KC532781 |
| 8_92 | KC529931 | KC530406 | KC530882 | KC531357 | KC531832 | KC532307 | KC532782 |
| 1605202443 | KC529932 | KC530407 | KC530883 | KC531358 | KC531833 | KC532308 | KC532783 |
| 1607001262 | KC529933 | KC530408 | KC530884 | KC531359 | KC531834 | KC532309 | KC532784 |
| 1608000352 | KC529934 | KC530409 | KC530885 | KC531360 | KC531835 | KC532310 | KC532785 |
| 1608000894 | KC529935 | KC530410 | KC530886 | KC531361 | KC531836 | KC532311 | KC532786 |
| 1609290340 | KC529936 | KC530411 | KC530887 | KC531362 | KC531837 | KC532312 | KC532787 |
| 1_488 | KC529937 | KC530412 | KC530888 | KC531363 | KC531838 | KC532313 | KC532788 |
| 1_489 | KC529938 | KC530413 | KC530889 | KC531364 | KC531839 | KC532314 | KC532789 |
| 1_587 | KC529939 | KC530414 | KC530890 | KC531365 | KC531840 | KC532315 | KC532790 |
| 1_588 | KC529940 | KC530415 | KC530891 | KC531366 | KC531841 | KC532316 | KC532791 |
| 1_846 | KC529941 | KC530416 | KC530892 | KC531367 | KC531842 | KC532317 | KC532792 |
| 109A | KC529942 | KC530417 | KC530893 | KC531368 | KC531843 | KC532318 | KC532793 |
| 109C | KC529943 | KC530418 | KC530894 | KC531369 | KC531844 | KC532319 | KC532794 |
| 110A | KC529944 | KC530419 | KC530895 | KC531370 | KC531845 | KC532320 | KC532795 |
| 110C | KC529945 | KC530420 | KC530896 | KC531371 | KC531846 | KC532321 | KC532796 |
| 110D | KC529946 | KC530421 | KC530897 | KC531372 | KC531847 | KC532322 | KC532797 |
| 110E | KC529947 | KC530422 | KC530898 | KC531373 | KC531848 | KC532323 | KC532798 |
| 11109 | KC529948 | KC530423 | KC530899 | KC531374 | KC531849 | KC532324 | KC532799 |
| 11112 | KC529949 | KC530424 | KC530900 | KC531375 | KC531850 | KC532325 | KC532800 |
| 1111I_08 | KC529950 | KC530425 | KC530901 | KC531376 | KC531851 | KC532326 | KC532801 |
| 1219 | KC529951 | KC530426 | KC530902 | KC531377 | KC531852 | KC532327 | KC532802 |
| 1291_09 | KC529952 | KC530427 | KC530903 | KC531378 | KC531853 | KC532328 | KC532803 |
| 130C | KC529953 | KC530428 | KC530904 | KC531379 | KC531854 | KC532329 | KC532804 |
| 130D | KC529954 | KC530429 | KC530905 | KC531380 | KC531855 | KC532330 | KC532805 |
| 189E | KC529955 | KC530430 | KC530906 | KC531381 | KC531856 | KC532331 | KC532806 |
| 20662_07 | KC529956 | KC530431 | KC530907 | KC531382 | KC531857 | KC532332 | KC532807 |
| 2461_07 | KC529957 | KC530432 | KC530908 | KC531383 | KC531858 | KC532333 | KC532808 |
| 2550II_07 | KC529958 | KC530433 | KC530909 | KC531384 | KC531859 | KC532334 | KC532809 |
| 2551_07 | KC529959 | KC530434 | KC530910 | KC531385 | KC531860 | KC532335 | KC532810 |
| 2551_07_CM | KC529960 | KC530435 | KC530911 | KC531386 | KC531861 | KC532336 | KC532811 |
| 269 | KC529961 | KC530436 | KC530912 | KC531387 | KC531862 | KC532337 | KC532812 |
| 28170_07 | KC529962 | KC530437 | KC530913 | KC531388 | KC531863 | KC532338 | KC532813 |
| 2895I_08 | KC529963 | KC530438 | KC530914 | KC531389 | KC531864 | KC532339 | KC532814 |
| 34 | KC529964 | KC530439 | KC530915 | KC531390 | KC531865 | KC532340 | KC532815 |
| 4_187 | KC529965 | KC530440 | KC530916 | KC531391 | KC531866 | KC532341 | KC532816 |
| 4_202 | KC529966 | KC530441 | KC530917 | KC531392 | KC531867 | KC532342 | KC532817 |
| 4_231 | KC529967 | KC530442 | KC530918 | KC531393 | KC531868 | KC532343 | KC532818 |
| 4_253 | KC529968 | KC530443 | KC530919 | KC531394 | KC531869 | KC532344 | KC532819 |
| 4_315 | KC529969 | KC530444 | KC530920 | KC531395 | KC531870 | KC532345 | KC532820 |
| 4_319 | KC529970 | KC530445 | KC530921 | KC531396 | KC531871 | KC532346 | KC532821 |
| 4_381 | KC529971 | KC530446 | KC530922 | KC531397 | KC531872 | KC532347 | KC532822 |
| 4_83 | KC529972 | KC530447 | KC530923 | KC531398 | KC531873 | KC532348 | KC532823 |
| 4_9 | KC529973 | KC530448 | KC530924 | KC531399 | KC531874 | KC532349 | KC532824 |
| 40 | KC529974 | KC530449 | KC530925 | KC531400 | KC531875 | KC532350 | KC532825 |
| 44 | KC529975 | KC530450 | KC530926 | KC531401 | KC531876 | KC532351 | KC532826 |
| 4500_07 | KC529976 | KC530451 | KC530927 | KC531402 | KC531877 | KC532352 | KC532827 |
| 50NC2 | KC529977 | KC530452 | KC530928 | KC531403 | KC531878 | KC532353 | KC532828 |
| 50NC5 | KC529978 | KC530453 | KC530929 | KC531404 | KC531879 | KC532354 | KC532829 |
| 96B | KC529979 | KC530454 | KC530930 | KC531405 | KC531880 | KC532355 | KC532830 |
| CN48 | KC529980 | KC530455 | KC530931 | KC531406 | KC531881 | KC532356 | KC532831 |
| CN49004 | KC529981 | KC530456 | KC530932 | KC531407 | KC531882 | KC532357 | KC532832 |
| CN49005 | KC529982 | KC530457 | KC530933 | KC531408 | KC531883 | KC532358 | KC532833 |
| CN49006 | KC529983 | KC530458 | KC530934 | KC531409 | KC531884 | KC532359 | KC532834 |
| CN49008 | KC529984 | KC530459 | KC530935 | KC531410 | KC531885 | KC532360 | KC532835 |
| CN4901 | KC529985 | KC530460 | KC530936 | KC531411 | KC531886 | KC532361 | KC532836 |
| CN4902 | KC529986 | KC530461 | KC530937 | KC531412 | KC531887 | KC532362 | KC532837 |
| CN4903 | KC529987 | KC530462 | KC530938 | KC531413 | KC531888 | KC532363 | KC532838 |
| CN4904 | KC529988 | KC530463 | KC530939 | KC531414 | KC531889 | KC532364 | KC532839 |
| CN4905 | KC529989 | KC530464 | KC530940 | KC531415 | KC531890 | KC532365 | KC532840 |
| CN4906 | KC529990 | KC530465 | KC530941 | KC531416 | KC531891 | KC532366 | KC532841 |
| CN4907 | KC529991 | KC530466 | KC530942 | KC531417 | KC531892 | KC532367 | KC532842 |
| CN4909 | KC529992 | KC530467 | KC530943 | KC531418 | KC531893 | KC532368 | KC532843 |
| CN4914 | KC529993 | KC530468 | KC530944 | KC531419 | KC531894 | KC532369 | KC532844 |
| CN4915 | KC529994 | KC530469 | KC530945 | KC531420 | KC531895 | KC532370 | KC532845 |
| CN4916 | KC529995 | KC530470 | KC530946 | KC531421 | KC531896 | KC532371 | KC532846 |
| CN4917 | KC529996 | KC530471 | KC530947 | KC531422 | KC531897 | KC532372 | KC532847 |
| CN4918 | KC529997 | KC530472 | KC530948 | KC531423 | KC531898 | KC532373 | KC532848 |
| CN4919 | KC529998 | KC530473 | KC530949 | KC531424 | KC531899 | KC532374 | KC532849 |
| CN4920 | KC529999 | KC530474 | KC530950 | KC531425 | KC531900 | KC532375 | KC532850 |
| CN4921 | KC530000 | KC530475 | KC530951 | KC531426 | KC531901 | KC532376 | KC532851 |
| CN4924 | KC530001 | KC530476 | KC530952 | KC531427 | KC531902 | KC532377 | KC532852 |
| CN4926 | KC530002 | KC530477 | KC530953 | KC531428 | KC531903 | KC532378 | KC532853 |
| CN4927 | KC530003 | KC530478 | KC530954 | KC531429 | KC531904 | KC532379 | KC532854 |
| CN4931 | KC530004 | KC530479 | KC530955 | KC531430 | KC531905 | KC532380 | KC532855 |
| CN4932 | KC530005 | KC530480 | KC530956 | KC531431 | KC531906 | KC532381 | KC532856 |
| CN4933 | KC530006 | KC530481 | KC530957 | KC531432 | KC531907 | KC532382 | KC532857 |
| CN4934 | KC530007 | KC530482 | KC530958 | KC531433 | KC531908 | KC532383 | KC532858 |
| CN4936 | KC530008 | KC530483 | KC530959 | KC531434 | KC531909 | KC532384 | KC532859 |
| CN4937 | KC530009 | KC530484 | KC530960 | KC531435 | KC531910 | KC532385 | KC532860 |
| CN4938 | KC530010 | KC530485 | KC530961 | KC531436 | KC531911 | KC532386 | KC532861 |
| CN4940 | KC530011 | KC530486 | KC530962 | KC531437 | KC531912 | KC532387 | KC532862 |
| CN4941 | KC530012 | KC530487 | KC530963 | KC531438 | KC531913 | KC532388 | KC532863 |
| CN4942 | KC530013 | KC530488 | KC530964 | KC531439 | KC531914 | KC532389 | KC532864 |
| CN4943 | KC530014 | KC530489 | KC530965 | KC531440 | KC531915 | KC532390 | KC532865 |
| CN4944 | KC530015 | KC530490 | KC530966 | KC531441 | KC531916 | KC532391 | KC532866 |
| CN4945 | KC530016 | KC530491 | KC530967 | KC531442 | KC531917 | KC532392 | KC532867 |
| CN4946 | KC530017 | KC530492 | KC530968 | KC531443 | KC531918 | KC532393 | KC532868 |
| CN4947 | KC530018 | KC530493 | KC530969 | KC531444 | KC531919 | KC532394 | KC532869 |
| CN4948 | KC530019 | KC530494 | KC530970 | KC531445 | KC531920 | KC532395 | KC532870 |
| CN4949 | KC530020 | KC530495 | KC530971 | KC531446 | KC531921 | KC532396 | KC532871 |
| CN4950 | KC530021 | KC530496 | KC530972 | KC531447 | KC531922 | KC532397 | KC532872 |
| CN4952 | KC530022 | KC530497 | KC530973 | KC531448 | KC531923 | KC532398 | KC532873 |
| CN4954 | KC530023 | KC530498 | KC530974 | KC531449 | KC531924 | KC532399 | KC532874 |
| CN4955 | KC530024 | KC530499 | KC530975 | KC531450 | KC531925 | KC532400 | KC532875 |
| CN4956 | KC530025 | KC530500 | KC530976 | KC531451 | KC531926 | KC532401 | KC532876 |
| CN4957 | KC530026 | KC530501 | KC530977 | KC531452 | KC531927 | KC532402 | KC532877 |
| CN4960 | KC530027 | KC530502 | KC530978 | KC531453 | KC531928 | KC532403 | KC532878 |
| CN4964 | KC530028 | KC530503 | KC530979 | KC531454 | KC531929 | KC532404 | KC532879 |
| CN4967 | KC530029 | KC530504 | KC530980 | KC531455 | KC531930 | KC532405 | KC532880 |
| CN4968 | KC530030 | KC530505 | KC530981 | KC531456 | KC531931 | KC532406 | KC532881 |
| CN4970 | KC530031 | KC530506 | KC530982 | KC531457 | KC531932 | KC532407 | KC532882 |
| CN4977 | KC530032 | KC530507 | KC530983 | KC531458 | KC531933 | KC532408 | KC532883 |
| CN4980 | KC530033 | KC530508 | KC530984 | KC531459 | KC531934 | KC532409 | KC532884 |
| CN4983 | KC530034 | KC530509 | KC530985 | KC531460 | KC531935 | KC532410 | KC532885 |
| CN4987 | KC530035 | KC530510 | KC530986 | KC531461 | KC531936 | KC532411 | KC532886 |
| CN4988 | KC530036 | KC530511 | KC530987 | KC531462 | KC531937 | KC532412 | KC532887 |
| CN4989 | KC530037 | KC530512 | KC530988 | KC531463 | KC531938 | KC532413 | KC532888 |
| CN4993 | KC530038 | KC530513 | KC530989 | KC531464 | KC531939 | KC532414 | KC532889 |
| CN4995 | KC530039 | KC530514 | KC530990 | KC531465 | KC531940 | KC532415 | KC532890 |
| CN4998 | KC530040 | KC530515 | KC530991 | KC531466 | KC531941 | KC532416 | KC532891 |
| CN5001 | KC530041 | KC530516 | KC530992 | KC531467 | KC531942 | KC532417 | KC532892 |
| CN5002 | KC530042 | KC530517 | KC530993 | KC531468 | KC531943 | KC532418 | KC532893 |
| CN5003 | KC530043 | KC530518 | KC530994 | KC531469 | KC531944 | KC532419 | KC532894 |
| CN5005 | KC530044 | KC530519 | KC530995 | KC531470 | KC531945 | KC532420 | KC532895 |
| CN5007 | KC530045 | KC530520 | KC530996 | KC531471 | KC531946 | KC532421 | KC532896 |
| CN5008 | KC530046 | KC530521 | KC530997 | KC531472 | KC531947 | KC532422 | KC532897 |
| CN5009 | KC530047 | KC530522 | KC530998 | KC531473 | KC531948 | KC532423 | KC532898 |
| CN5010 | KC530048 | KC530523 | KC530999 | KC531474 | KC531949 | KC532424 | KC532899 |
| CN5011 | KC530049 | KC530524 | KC531000 | KC531475 | KC531950 | KC532425 | KC532900 |
| CN5012 | KC530050 | KC530525 | KC531001 | KC531476 | KC531951 | KC532426 | KC532901 |
| CN5013 | KC530051 | KC530526 | KC531002 | KC531477 | KC531952 | KC532427 | KC532902 |
| CN5014 | KC530052 | KC530527 | KC531003 | KC531478 | KC531953 | KC532428 | KC532903 |
| CN5015 | KC530053 | KC530528 | KC531004 | KC531479 | KC531954 | KC532429 | KC532904 |
| CN5017 | KC530054 | KC530529 | KC531005 | KC531480 | KC531955 | KC532430 | KC532905 |
| CN5018 | KC530055 | KC530530 | KC531006 | KC531481 | KC531956 | KC532431 | KC532906 |
| CN5019 | KC530056 | KC530531 | KC531007 | KC531482 | KC531957 | KC532432 | KC532907 |
| D1 | KC530057 | KC530532 | KC531008 | KC531483 | KC531958 | KC532433 | KC532908 |
| D12 | KC530058 | KC530533 | KC531009 | KC531484 | KC531959 | KC532434 | KC532909 |
| D14 | KC530059 | KC530534 | KC531010 | KC531485 | KC531960 | KC532435 | KC532910 |
| D15 | KC530060 | KC530535 | KC531011 | KC531486 | KC531961 | KC532436 | KC532911 |
| D16 | KC530061 | KC530536 | KC531012 | KC531487 | KC531962 | KC532437 | KC532912 |
| D17 | KC530062 | KC530537 | KC531013 | KC531488 | KC531963 | KC532438 | KC532913 |
| D18 | KC530063 | KC530538 | KC531014 | KC531489 | KC531964 | KC532439 | KC532914 |
| D19 | KC530064 | KC530539 | KC531015 | KC531490 | KC531965 | KC532440 | KC532915 |
| D2 | KC530065 | KC530540 | KC531016 | KC531491 | KC531966 | KC532441 | KC532916 |
| D21 | KC530066 | KC530541 | KC531017 | KC531492 | KC531967 | KC532442 | KC532917 |
| D22 | KC530067 | KC530542 | KC531018 | KC531493 | KC531968 | KC532443 | KC532918 |
| D26 | KC530068 | KC530543 | KC531019 | KC531494 | KC531969 | KC532444 | KC532919 |
| D27 | KC530069 | KC530544 | KC531020 | KC531495 | KC531970 | KC532445 | KC532920 |
| D28 | KC530070 | KC530545 | KC531021 | KC531496 | KC531971 | KC532446 | KC532921 |
| D3 | KC530071 | KC530546 | KC531022 | KC531497 | KC531972 | KC532447 | KC532922 |
| D30 | KC530072 | KC530547 | KC531023 | KC531498 | KC531973 | KC532448 | KC532923 |
| D31 | KC530073 | KC530548 | KC531024 | KC531499 | KC531974 | KC532449 | KC532924 |
| D33 | KC530074 | KC530549 | KC531025 | KC531500 | KC531975 | KC532450 | KC532925 |
| D34 | KC530075 | KC530550 | KC531026 | KC531501 | KC531976 | KC532451 | KC532926 |
| D35 | KC530076 | KC530551 | KC531027 | KC531502 | KC531977 | KC532452 | KC532927 |
| D36 | KC530077 | KC530552 | KC531028 | KC531503 | KC531978 | KC532453 | KC532928 |
| D4 | KC530078 | KC530553 | KC531029 | KC531504 | KC531979 | KC532454 | KC532929 |
| D41 | KC530079 | KC530554 | KC531030 | KC531505 | KC531980 | KC532455 | KC532930 |
| D42 | KC530080 | KC530555 | KC531031 | KC531506 | KC531981 | KC532456 | KC532931 |
| D43 | KC530081 | KC530556 | KC531032 | KC531507 | KC531982 | KC532457 | KC532932 |
| D44 | KC530082 | KC530557 | KC531033 | KC531508 | KC531983 | KC532458 | KC532933 |
| D45 | KC530083 | KC530558 | KC531034 | KC531509 | KC531984 | KC532459 | KC532934 |
| D46 | KC530084 | KC530559 | KC531035 | KC531510 | KC531985 | KC532460 | KC532935 |
| D6 | KC530085 | KC530560 | KC531036 | KC531511 | KC531986 | KC532461 | KC532936 |
| D64 | KC530086 | KC530561 | KC531037 | KC531512 | KC531987 | KC532462 | KC532937 |
| D69 | KC530087 | KC530562 | KC531038 | KC531513 | KC531988 | KC532463 | KC532938 |
| D71 | KC530088 | KC530563 | KC531039 | KC531514 | KC531989 | KC532464 | KC532939 |
| D73 | KC530089 | KC530564 | KC531040 | KC531515 | KC531990 | KC532465 | KC532940 |
| D76 | KC530090 | KC530565 | KC531041 | KC531516 | KC531991 | KC532466 | KC532941 |
| D9 | KC530091 | KC530566 | KC531042 | KC531517 | KC531992 | KC532467 | KC532942 |
| P6 | KC530092 | KC530567 | KC531043 | KC531518 | KC531993 | KC532468 | KC532943 |
| PG1 | KC530093 | KC530568 | KC531044 | KC531519 | KC531994 | KC532469 | KC532944 |
| PG2 | KC530094 | KC530569 | KC531045 | KC531520 | KC531995 | KC532470 | KC532945 |
| PG21 | KC530095 | KC530570 | KC531046 | KC531521 | KC531996 | KC532471 | KC532946 |
| PG26 | KC530096 | KC530571 | KC531047 | KC531522 | KC531997 | KC532472 | KC532947 |
| PG3 | KC530097 | KC530572 | KC531048 | KC531523 | KC531998 | KC532473 | KC532948 |
| PG32 | KC530098 | KC530573 | KC531049 | KC531524 | KC531999 | KC532474 | KC532949 |
| PG37 | KC530099 | KC530574 | KC531050 | KC531525 | KC532000 | KC532475 | KC532950 |
| PG46 | KC530100 | KC530575 | KC531051 | KC531526 | KC532001 | KC532476 | KC532951 |
| CM1 | KC530101 | KC530576 | KC531052 | KC531527 | KC532002 | KC532477 | KC532952 |
| CM10 | KC530102 | KC530577 | KC531053 | KC531528 | KC532003 | KC532478 | KC532953 |
| CM11 | KC530103 | KC530578 | KC531054 | KC531529 | KC532004 | KC532479 | KC532954 |
| CM12 | KC530104 | KC530579 | KC531055 | KC531530 | KC532005 | KC532480 | KC532955 |
| CM13 | KC530105 | KC530580 | KC531056 | KC531531 | KC532006 | KC532481 | KC532956 |
| CM14 | KC530106 | KC530581 | KC531057 | KC531532 | KC532007 | KC532482 | KC532957 |
| CM15 | KC530107 | KC530582 | KC531058 | KC531533 | KC532008 | KC532483 | KC532958 |
| CM16 | KC530108 | KC530583 | KC531059 | KC531534 | KC532009 | KC532484 | KC532959 |
| CM17 | KC530109 | KC530584 | KC531060 | KC531535 | KC532010 | KC532485 | KC532960 |
| CM18 | KC530110 | KC530585 | KC531061 | KC531536 | KC532011 | KC532486 | KC532961 |
| CM2 | KC530111 | KC530586 | KC531062 | KC531537 | KC532012 | KC532487 | KC532962 |
| CM20 | KC530112 | KC530587 | KC531063 | KC531538 | KC532013 | KC532488 | KC532963 |
| CM22 | KC530113 | KC530588 | KC531064 | KC531539 | KC532014 | KC532489 | KC532964 |
| CM23 | KC530114 | KC530589 | KC531065 | KC531540 | KC532015 | KC532490 | KC532965 |
| CM24 | KC530115 | KC530590 | KC531066 | KC531541 | KC532016 | KC532491 | KC532966 |
| CM25 | KC530116 | KC530591 | KC531067 | KC531542 | KC532017 | KC532492 | KC532967 |
| CM26 | KC530117 | KC530592 | KC531068 | KC531543 | KC532018 | KC532493 | KC532968 |
| CM27 | KC530118 | KC530593 | KC531069 | KC531544 | KC532019 | KC532494 | KC532969 |
| CM28 | KC530119 | KC530594 | KC531070 | KC531545 | KC532020 | KC532495 | KC532970 |
| CM29 | KC530120 | KC530595 | KC531071 | KC531546 | KC532021 | KC532496 | KC532971 |
| CM3 | KC530121 | KC530596 | KC531072 | KC531547 | KC532022 | KC532497 | KC532972 |
| CM30 | KC530122 | KC530597 | KC531073 | KC531548 | KC532023 | KC532498 | KC532973 |
| CM32 | KC530123 | KC530598 | KC531074 | KC531549 | KC532024 | KC532499 | KC532974 |
| CM33 | KC530124 | KC530599 | KC531075 | KC531550 | KC532025 | KC532500 | KC532975 |
| CM34 | KC530125 | KC530600 | KC531076 | KC531551 | KC532026 | KC532501 | KC532976 |
| CM35 | KC530126 | KC530601 | KC531077 | KC531552 | KC532027 | KC532502 | KC532977 |
| CM36 | KC530127 | KC530602 | KC531078 | KC531553 | KC532028 | KC532503 | KC532978 |
| CM37 | KC530128 | KC530603 | KC531079 | KC531554 | KC532029 | KC532504 | KC532979 |
| CM38 | KC530129 | KC530604 | KC531080 | KC531555 | KC532030 | KC532505 | KC532980 |
| CM39 | KC530130 | KC530605 | KC531081 | KC531556 | KC532031 | KC532506 | KC532981 |
| CM4 | KC530131 | KC530606 | KC531082 | KC531557 | KC532032 | KC532507 | KC532982 |
| CM40 | KC530132 | KC530607 | KC531083 | KC531558 | KC532033 | KC532508 | KC532983 |
| CM41 | KC530133 | KC530608 | KC531084 | KC531559 | KC532034 | KC532509 | KC532984 |
| CM42 | KC530134 | KC530609 | KC531085 | KC531560 | KC532035 | KC532510 | KC532985 |
| CM43 | KC530135 | KC530610 | KC531086 | KC531561 | KC532036 | KC532511 | KC532986 |
| CM44 | KC530136 | KC530611 | KC531087 | KC531562 | KC532037 | KC532512 | KC532987 |
| CM45 | KC530137 | KC530612 | KC531088 | KC531563 | KC532038 | KC532513 | KC532988 |
| CM46 | KC530138 | KC530613 | KC531089 | KC531564 | KC532039 | KC532514 | KC532989 |
| CM47 | KC530139 | KC530614 | KC531090 | KC531565 | KC532040 | KC532515 | KC532990 |
| CM48 | KC530140 | KC530615 | KC531091 | KC531566 | KC532041 | KC532516 | KC532991 |
| CM49 | KC530141 | KC530616 | KC531092 | KC531567 | KC532042 | KC532517 | KC532992 |
| CM5 | KC530142 | KC530617 | KC531093 | KC531568 | KC532043 | KC532518 | KC532993 |
| CM50 | KC530143 | KC530618 | KC531094 | KC531569 | KC532044 | KC532519 | KC532994 |
| CM51 | KC530144 | KC530619 | KC531095 | KC531570 | KC532045 | KC532520 | KC532995 |
| CM52 | KC530145 | KC530620 | KC531096 | KC531571 | KC532046 | KC532521 | KC532996 |
| CM55 | KC530146 | KC530621 | KC531097 | KC531572 | KC532047 | KC532522 | KC532997 |
| CM56 | KC530147 | KC530622 | KC531098 | KC531573 | KC532048 | KC532523 | KC532998 |
| CM57 | KC530148 | KC530623 | KC531099 | KC531574 | KC532049 | KC532524 | KC532999 |
| CM58 | KC530149 | KC530624 | KC531100 | KC531575 | KC532050 | KC532525 | KC533000 |
| CM59 | KC530150 | KC530625 | KC531101 | KC531576 | KC532051 | KC532526 | KC533001 |
| CM6 | KC530151 | KC530626 | KC531102 | KC531577 | KC532052 | KC532527 | KC533002 |
| CM60 | KC530152 | KC530627 | KC531103 | KC531578 | KC532053 | KC532528 | KC533003 |
| CM61 | KC530153 | KC530628 | KC531104 | KC531579 | KC532054 | KC532529 | KC533004 |
| CM63 | KC530154 | KC530629 | KC531105 | KC531580 | KC532055 | KC532530 | KC533005 |
| CM64 | KC530155 | KC530630 | KC531106 | KC531581 | KC532056 | KC532531 | KC533006 |
| CM7 | KC530156 | KC530631 | KC531107 | KC531582 | KC532057 | KC532532 | KC533007 |
| CM8 | KC530157 | KC530632 | KC531108 | KC531583 | KC532058 | KC532533 | KC533008 |

Table D. Allele types (ATs) of each locus and Sequence Type (STs).

| ***CAP59*** | ***GPD1*** | **IGS1** | ***LAC1*** | ***PLB1*** | ***SOD1*** | ***URA5*** | **ST** |
| --- | --- | --- | --- | --- | --- | --- | --- |
| 1 | 1 | 1 | 4 | 2 | 1 | 5 | 4 |
| 1 | 3 | 1 | 5 | 2 | 1 | 1 | 5 |
| 1 | 1 | 1 | 3 | 2 | 1 | 5 | 6 |
| 7 | 1 | 1 | 2 | 1 | 1 | 2 | 23 |
| 1 | 1 | 10 | 3 | 2 | 1 | 1 | 31 |
| 2 | 9 | 14 | 8 | 11 | 12 | 4 | 40 |
| 1 | 3 | 1 | 3 | 2 | 1 | 1 | 53 |
| 7 | 5 | 1 | 3 | 3 | 1 | 1 | 69 |
| 7 | 5 | 1 | 3 | 4 | 1 | 1 | 71 |
| 1 | 1 | 25 | 3 | 2 | 1 | 1 | 77 |
| 1 | 1 | 1 | 9 | 2 | 1 | 5 | 82 |
| 1 | 23 | 10 | 3 | 4 | 1 | 1 | 93 |
| 1 | 1 | 1 | 4 | 2 | 1 | 14 | 141 |
| 1 | 1 | 1 | 3 | 2 | 1 | 1 | 174 |
| 1 | 1 | 1 | 4 | 2 | 1 | 1 | 175 |
| 1 | 23 | 1 | 3 | 2 | 1 | 5 | 176 |
| 1 | 23 | 10 | 3 | 4 | 1 | 5 | 177 |
| 1 | 1 | 10 | 3 | 2 | 36 | 1 | 185 |
| 1 | 26 | 1 | 5 | 2 | 1 | 1 | 186 |
| 1 | 23 | 10 | 3 | 2 | 1 | 1 | 187 |
| 1 | 1 | 1 | 5 | 2 | 1 | 5 | 188 |
| 1 | 1 | 1 | 7 | 2 | 1 | 5 | 189 |
| 1 | 1 | 10 | 4 | 2 | 1 | 5 | 190 |
| 1 | 1 | 10 | 5 | 2 | 1 | 1 | 191 |
| 1 | 1 | 59 | 3 | 2 | 1 | 1 | 192 |
| 1 | 3 | 1 | 5 | 2 | 1 | 5 | 193 |
| 1 | 3 | 10 | 5 | 2 | 1 | 1 | 194 |
| 1 | 23 | 10 | 5 | 4 | 1 | 1 | 195 |

Table E. DNA polymorphisms of Asian *C*. *neoformans* var. *grubii* from different locations.

| **Location** | | **S** | **π** | **θ_s_** | ***k*** | ***h*** | ***H_d_*** | ***D*** | ***F_D_*** | ***F_F_*** | ***F_s_*** |
| --- | --- | --- | --- | --- | --- | --- | --- | --- | --- | --- | --- |
| East Asia | | 20 | 0.0004 | 3.636 | 1.415 | 11 | 0.205 | -1.702 | -0.638 | -1.256 | -2.607 |
|  | China | 14 | 0.0004 | 2.786 | 1.668 | 8 | 0.258 | -1.120 | 1.550* | -0.710 | -0.336 |
|  | Hong Kong | 4 | 0.0002 | 1.258 | 0.934 | 3 | 0.275 | -0.848 | 0.305 | 0.004 | 0.711 |
|  | Japan | 17 | 0.0003 | 4.046 | 0.994 | 3 | 0.104 | -2.468** | -4.180** | -4.268** | 1.825 |
| South/Southeast Asia | | 75 | 0.0015 | 11.816 | 6.191 | 21 | 0.779 | -1.401 | -8.820** | -6.351** | 1.794 |
|  | India | 65 | 0.0015 | 13.889 | 5.945 | 10 | 0.699 | -1.951* | -5.205** | -4.742** | 3.453 |
|  | Indonesia | 20 | 0.0019 | 4.702 | 7.564 | 5 | 0.717 | 2.013 | 1.662** | 2.104** | 10.344 |
|  | Thailand | 23 | 0.0005 | 3.854 | 1.984 | 14 | 0.661 | -1.314 | -1.484 | -1.704 | -2.243 |
| Middle East | | 26 | 0.0020 | 7.996 | 7.962 | 10 | 0.924 | -0.018 | -0.267 | -0.228 | -0.658 |
|  | Kuwait | 26 | 0.0021 | 9.191 | 8.533 | 9 | 0.978 | -0.341 | -0.122 | -0.199 | -2.020 |
|  | Qatar | 14 | 0.0020 | 6.720 | 8.000 | 3 | 0.800 | 1.388 | 1.388 | 1.477 | 3.297 |

S: number of polymorphic sites

π: nucleotide diversity

θ_s_: Watterson’s mutation rate per sequence

*k*: average number of nucleotide differences per sequence

h: number of haplotypes

*H_d_*: haplotype diversity

*D*, *F* and *F_s_*: Tajima’s D, Fu and Li’s F* and Fu’s Fs, respectively. The p value <0.05, *; <0.01, **.

Table F. DNA polymorphisms of *C*. *neoformans* var. *grubii* from different continents.

| **Location** | **S** | **π** | **θ_s_** | ***k*** | ***h*** | ***H_d_*** | ***D*** | ***F_D_*** | ***F_F_*** | ***F_s_*** |
| --- | --- | --- | --- | --- | --- | --- | --- | --- | --- | --- |
| Africa | 124 | 0.0062 | 28.966 | 24.510 | 34 | 0.988 | -0.559 | -1.365 | -1.278 | -5.569 |
| North/South America | 79 | 0.0067 | 19.775 | 26.658 | 16 | 0.927 | 1.314 | 1.342 | 1.576 | 4.070 |
| Asia | 81 | 0.0016 | 11.830 | 6.232 | 32 | 0.780 | -1.353 | 0.916 | -0.252 | -1.162 |
| Europe | 78 | 0.0025 | 17.576 | 9.788 | 17 | 0.841 | -1.570 | -1.037 | -1.472 | 0.673 |

S: number of polymorphic sites

π: nucleotide diversity

θ_s_: Watterson’s mutation rate per sequence

*k*: average number of nucleotide differences per sequence

h: number of haplotypes

*H_d_*: haplotype diversity

*D*, *F* and *F_s_*: Tajima’s D, Fu and Li’s F* and Fu’s Fs, respectively. The *p*-values of these statistics were not significant (*p*>0.05).

Table G. Genetic differentiation amongst populations from different Asian regions in pairwise comparison.

| **Population I** | **Population II** | ***F_ST_*** | ***Nm*** |
| --- | --- | --- | --- |
| East Asia | South/Southeast Asia | 0.351*** | 0.93 |
| East Asia | Middle East | 0.233*** | 1.64 |
| South/Southeast Asia | Middle East | 0.019*** | 25.76 |

*F_ST_* : Wright’s fixation index

Nm: Gene flow estimate values

***: *p<*0.001

Table H. Genetic differentiation amongst populations from different continents in pairwise comparison.

| **Population I** | **Population II** | ***F_ST_*** | ***Nm*** |
| --- | --- | --- | --- |
| Africa | North/South America | 0.116** | 3.81 |
| Africa | Asia | 0.193*** | 2.09 |
| Africa | Europe | 0.176*** | 2.34 |
| North/South America | Asia | 0.194*** | 2.08 |
| North/South America | Europe | 0.150*** | 2.84 |
| Asia | Europe | 0.222*** | 1.76 |

*F_ST_* : Wright’s fixation index

Nm: Gene flow estimate values

**:*p*<0.01

***: *p*<0.001

Table I. Pairwise linkage disequilibrium of Asian *C*. *neoformans* according to seven MLST loci and geographical origin.

| Locus | Country | Zns | Pairwise | Fisher | Bonferroni | Rm |
| --- | --- | --- | --- | --- | --- | --- |
| *CAP59* |  | 0.67 | 15 | 10 | 10 | 0 |
|  | China | ND | 0 | 0 | 10 | ND |
|  | Hong Kong | ND | 0 | 0 | 10 | ND |
|  | India | 0.67 | 15 | 10 | 0 | 0 |
|  | Indonesia | ND | 0 | 0 | 0 | 0 |
|  | Japan | ND | 0 | 0 | 0 | 0 |
|  | Middle East | ND | 0 | 0 | 0 | 0 |
|  | Thailand | ND | 0 | 0 | 0 | ND |
| *GPD1* |  | 0.34 | 55 | 25 | 4 | 1 |
|  | China | 0.31 | 1 | 1 | 1 | 1 |
|  | Hong Kong | ND | 0 | 0 | 1 | 0 |
|  | India | 0.45 | 45 | 24 | 0 | 0 |
|  | Indonesia | 0.53 | 6 | 3 | 3 | 0 |
|  | Japan | ND | 0 | 0 | 3 | 0 |
|  | Middle East | 0.31 | 10 | 0 | 0 | 0 |
|  | Thailand | 0.003 | 1 | 0 | 0 | 0 |
| IGS1 |  | 0.45 | 120 | 72 | 66 | 1 |
|  | China | 1.00 | 45 | 45 | 45 | 0 |
|  | Hong Kong | ND | 0 | 0 | 45 | ND |
|  | India | 0.43 | 120 | 52 | 46 | 0 |
|  | Indonesia | 1.00 | 45 | 45 | 45 | 0 |
|  | Japan | 1.00 | 45 | 45 | 0 | 0 |
|  | Middle East | 0.95 | 45 | 45 | 36 | 0 |
|  | Thailand | 1.00 | 45 | 45 | 45 | 0 |
| *LAC1* |  | 0.19 | 105 | 24 | 1 | 0 |
|  | China | ND | 0 | 0 | 1 | 0 |
|  | Hong Kong | 0.46 | 1 | 0 | 0 | 0 |
|  | India | 0.39 | 36 | 20 | 1 | 0 |
|  | Indonesia | ND | 0 | 0 | 1 | 0 |
|  | Japan | 0.49 | 1 | 0 | 0 | 0 |
|  | Middle East | 0.04 | 3 | 0 | 0 | 0 |
|  | Thailand | 0.48 | 21 | 11 | 1 | 0 |
| *PLB1* |  | 0.36 | 45 | 17 | 1 | 0 |
|  | China | ND | 0 | 0 | 1 | 0 |
|  | Hong Kong | ND | 0 | 0 | 1 | ND |
|  | India | 0.72 | 21 | 15 | 0 | 0 |
|  | Indonesia | 0.37 | 3 | 1 | 1 | 0 |
|  | Japan | 1.00 | 1 | 1 | 1 | 0 |
|  | Middle East | 0.25 | 6 | 0 | 0 | 0 |
|  | Thailand | ND | 0 | 0 | 0 | 0 |
| *SOD1* |  | 0.82 | 55 | 45 | 0 | 0 |
|  | China | ND | 0 | 0 | 0 | ND |
|  | Hong Kong | ND | 0 | 0 | 0 | ND |
|  | India | 1.00 | 45 | 45 | 0 | 0 |
|  | Indonesia | ND | 0 | 0 | 0 | ND |
|  | Japan | ND | 0 | 0 | 0 | ND |
|  | Middle East | ND | 0 | 0 | 0 | 0 |
|  | Thailand | ND | 0 | 0 | 0 | 0 |
| *URA5* |  | 0.51 | 55 | 29 | 0 | 0 |
|  | China | ND | 0 | 0 | 0 | 0 |
|  | Hong Kong | ND | 0 | 0 | 0 | 0 |
|  | India | 0.62 | 45 | 28 | 0 | 0 |
|  | Indonesia | 0.05 | 1 | 0 | 0 | 0 |
|  | Japan | ND | 0 | 0 | 0 | 0 |
|  | Middle East | 0.02 | 1 | 0 | 0 | 0 |
|  | Thailand | 0.0009 | 1 | 0 | 0 | 0 |

Zns : ZnS statistic (Kelly, 1997); the average of R^2^ (Hill and Robertson, 1986) of all pairwise comparisons.

Pairwise : Number of pairwise comparisons

Fisher : Number of significant pairwise comparisons by Fisher’s exact test (*p*<0.05)

Bonferroni : Number of significant comparison by Boferroni method (*p*<0.05)

Rm : Minimum number of recombination events

Table J. The Pairwise Homoplasy Index (PHI) test for recombination of Asian *C*. *neoformans* according to seven MLST loci and geographical origin.

| **Locations** | **Locus** | **Variance** | **Observed** | ***p*-value** |
| --- | --- | --- | --- | --- |
| East Asia | Concatenated | 0.005 | 0.286 | 0.014 |
|  | CAP59 | ND | ND | ND |
|  | GPD1 | NaN | 1 | 1 |
|  | IGS1 | 0 | 0 | 1 |
|  | LAC1 | ND | ND | ND |
|  | PLB1 | ND | ND | ND |
|  | SOD1 | ND | ND | ND |
|  | URA5 | ND | ND | ND |
| Middle East | Concatenated | 0.002 | 0.063 | 0.255 |
|  | CAP59 | ND | ND | ND |
|  | GPD1 | ND | ND | ND |
|  | IGS1 | 0 | 0 | 1 |
|  | LAC1 | NaN | 0 | 1 |
|  | PLB1 | ND | ND | ND |
|  | SOD1 | ND | ND | ND |
|  | URA5 | NaN | 0 | 1 |
| South/Southeast Asia | Concatenated | 0.004 | 0.04 | 8.01x10^-4^ |
|  | CAP59 | 0 | 0 | 1 |
|  | GPD1 | 0 | 0 | 1 |
|  | IGS1 | 0 | 0 | 1 |
|  | LAC1 | 0 | 0 | 1 |
|  | PLB1 | NaN | 0 | 1 |
|  | SOD1 | ND | ND | ND |
|  | URA5 | NaN | 0 | 1 |
| Global | Concatenated | 3.501 | 0.06 | 3.25x10^-14^ |
|  | CAP59 | 0.001 | 0 | 0.199 |
|  | GPD1 | 1.535 | 0 | 0.221 |
|  | IGS1 | 7.401 | 0.091 | 0.452 |
|  | LAC1 | NaN | 0 | 1 |
|  | PLB1 | 4.988 | 0 | 0.07 |
|  | SOD1 | 4.189 | 0.085 | 0.813 |
|  | URA5 | 1.481 | 0 | 0.143 |

ND : Not determined

NaN : Not a number/invalid
